# Fluctuating Nonlinear Spring Model of Mechanical Deformation of Biological Particles

**DOI:** 10.1371/journal.pcbi.1004729

**Published:** 2016-01-28

**Authors:** Olga Kononova, Joost Snijder, Yaroslav Kholodov, Kenneth A. Marx, Gijs J. L. Wuite, Wouter H. Roos, Valeri Barsegov

**Affiliations:** 1 Department of Chemistry, University of Massachusetts, Lowell, Massachusetts, United States of America; 2 Moscow Institute of Physics and Technology, Moscow Region, Russia; 3 Natuur- en Sterrenkunde and LaserLab, Vrije Universiteit, Amsterdam, The Netherlands; 4 Institute of Computer Aided Design Russian Academy of Science, Moscow, Russia; 5 Moleculaire Biofysica, Zernike instituut, Rijksuniversiteit Groningen, Groningen, The Netherlands; Rutgers University, UNITED STATES

## Abstract

The mechanical properties of virus capsids correlate with local conformational dynamics in the capsid structure. They also reflect the required stability needed to withstand high internal pressures generated upon genome loading and contribute to the success of important events in viral infectivity, such as capsid maturation, genome uncoating and receptor binding. The mechanical properties of biological nanoparticles are often determined from monitoring their dynamic deformations in Atomic Force Microscopy nanoindentation experiments; but a comprehensive theory describing the full range of observed deformation behaviors has not previously been described. We present a new theory for modeling dynamic deformations of biological nanoparticles, which considers the non-linear Hertzian deformation, resulting from an indenter-particle physical contact, and the bending of curved elements (beams) modeling the particle structure. The beams’ deformation beyond the critical point triggers a dynamic transition of the particle to the collapsed state. This extreme event is accompanied by a catastrophic force drop as observed in the experimental or simulated force (*F*)-deformation (*X*) spectra. The theory interprets fine features of the spectra, including the nonlinear components of the *FX*-curves, in terms of the Young’s moduli for Hertzian and bending deformations, and the structural damage dependent beams’ survival probability, in terms of the maximum strength and the cooperativity parameter. The theory is exemplified by successfully describing the deformation dynamics of natural nanoparticles through comparing theoretical curves with experimental force-deformation spectra for several virus particles. This approach provides a comprehensive description of the dynamic structural transitions in biological and artificial nanoparticles, which is essential for their optimal use in nanotechnology and nanomedicine applications.

## Author Summary

Dynamic force experiments, which have become available to explore the physical properties of biological assemblies, oftentimes reveal results that are difficult to understand without theoretical framework. We employed a multiscale modeling approach—a combination of Molecular Dynamics simulations of atomic structures with Langevin simulations of coarse-grained models of virus shells—to characterize the degrees of freedom defining the deformation and structural collapse of biological particles tested mechanically. This enabled us to develop an analytical model that provides meaningful interpretation of force-deformation spectra available from single-particle nanoindentation experiments. The Fluctuating Nonlinear Spring (FNS) model of uniaxial particle’s deformation captures essential features of the force-deformation spectra as observed in nanomanipulations *in vitro* and *in silico*: initial non-linearity, then a subsequent force decrease transition due to structural collapse. Our theory uniquely combines the elements of continuum mechanics with the statistics of extremes, enabling one to gather mechanical and statistical characteristics of nanoparticles, which determine the Hertzian deformation of the particle’s protein layer, and bending deformation and structural damage to the particle structure. We have demonstrated how the FNS theory can accurately model the deformation of several viral shells, showing promising model applications for describing a variety of natural and synthetic nanoparticles.

## Introduction

Single-molecule techniques, such as Atomic Force Microscopy (AFM), have become widely available to explore the physical properties of biological assemblies. These techniques have triggered extensive research efforts to explore the protein shells of plant and animal viruses, and bacteriophages. A wide spectrum of viruses infect their animal and plant hosts. To do so, these pathogens employ a diverse range of infection mechanisms. Typically, animal cells utilize a mechanism involving molecular recognition of the host via specific cell surface receptors. On the other hand, plant viruses often lack this host infectivity mechanism. In contrast to animal cells, plant cells are enclosed within a rigid cell wall and cuticle. These features represent significant physical barriers to viral infection. Thus, it is thought that physical damage to a plant’s surface that exposes the underlying cells, often through mechanical stress or as a result of insect feeding, is a requirement for viral infection to occur. Regardless of these viral infectivity mechanism differences, dynamic structural transitions by the viral capsids appear to be general features of most viruses. These infectivity mechanism differences are very likely correlated with variable virus capsids’ structures, dynamics and energetics-based mechanical properties. Therefore, understanding viral infectivity represents a clear motivation for investigating capsids’ mechanical and dynamic property differences.

The AFM-based mechanical testing of viral nanoparticles has now become the principal tool to probe the physico-chemical and materials properties of viruses [1]. In these experiments, an indenter (cantilever tip) approaches a particle and gradually deforms the particle, while the restoring (indentation) force *F* from the particle, corresponding to the particle deformation *X*, is measured. A variety of viruses have been characterized by profiling *F* as a function of *X* (*FX*-curve), including bacteriophages Φ29 and *HK*97 [2–4], the human viruses Noro Virus, Hepatitis B Virus, Human Immuno Deficiency Virus (HIV), Adenovirus (AdV) and Herpes Simplex Virus [5–9], and other eukaryotic cell infecting viruses such as Minute Virus of Mice, Triatoma Virus (TrV) and plant viruses Cowpea Chlorotic Mottle Virus (CCMV) and Brome Mosaic Virus (BMV) [10–14]. The *FX*-curves reveal valuable information about the particle spring constant, reversibility of deformation, and forces required to deform or distort capsid structures tested mechanically.

AFM experiments reveal a surprising diversity of mechanical properties of biological particles. These properties have been shown to correlate with local conformational dynamics of the capsid structure and to contribute to events such as receptor binding, genome uncoating and capsid maturation, all crucial steps in different viral infectious cycles. The main impediment to gaining further energetic and structural insights into these properties is that experiments reveal results that are difficult to interpret without a comprehensive theoretical modeling framework that describes the full range of observed mechanical behaviours. For example, it is not clear why is the initial portion of the *FX* spectra is weakly non-linear? Why do the *FX* spectra for some particles exhibit sudden drops in the deformation force, whereas the *FX* curves for other particles show gradual force decreases? What features determine the mechanical limits of the particle, i.e. the critical forces and critical deformations? Why do the *FX* spectra differ from one measurement to another for the same particle, even when it is indented along the same symmetry axis? The latter property points to the stochastic nature of deformation and collapse transitions, but what defines the likelihood of structural collapse at a given force load? Virus particles are often characterized by their spring constants, but our *in silico* nanoindentation studies show that the derivative, *dF*/*dX*, fluctuates significantly with *X* [15]. What is the extent of structure remodeling that gives rise to a non-monotonic behavior for *dF*/*dX*? What types of mechanical excitations corresponding to these structure alterations contribute to the particle deformation? These questions clearly show the need for a thorough theoretical framework describing capsid and other types of nanoshell deformations.

A number of theoretical approaches have been designed to describe the dynamics of virus particles, including: finite element analysis [16], normal mode analysis [17], elastic network modeling [18], atomistic MD and coarse-grained simulations [19–22], and other approaches [23]. Building upon the results from direct MD simulations of mechanical deformation, here we take a step further to develop a systematic approach for meaningful interpretation of the force-deformation spectral lineshapes available from single-particle nanomanipulation experiments. In these state-of-the-art experiments, a slowly moving cantilever tip gradually deforms a biological particle, and multiple nanoindentations are performed to directly probe the particle’s mechanical response. Using slow indenter velocities is entirely justified biologically. This view can be seen to align with the kinetics of genome packaging and ejection, which occur on a second timescale or shorter, as does the associated pressure change occurring inside the particle. For these reasons, we formulate a theoretical model for a uniaxial particle’s deformation achieved using slow indenter velocities. The theory links the slope, critical force, and the critical deformation of the *FX*-curve with the physical characteristics of the structure, geometry and overall shape of the particle and indenter. First, we summarize the results of Molecular Dynamics (MD) simulations of mechanical deformation accelerated on Graphics Processing Units (GPUs) [24, 25], which we refer to as nanoindentation *in silico*, of the empty CCMV capsid particle; see S1 Fig [15]. The in-depth analysis of the structure and energy output from MD simulations for this specific example of a thick-shelled nanoparticle has enabled us to identify the most important types of mechanical excitations that contribute to the deformation of biological particles. Next, we formulate the model by analyzing structural evidence from *in silico* nanoindentation measurements, which mimic the nanoindentation experiments *in vitro*. Finally, we apply the model to characterize the experimental and simulated *FX*-spectra for several specific examples of biological nanoparticles: the protein shells of the viruses CCMV, AdV and TrV.

## Results

### Nanoindentation *in silico*

We employed the methodology of “nanoindentation *in silico*” (i.e. computational-based indentation of a nanoparticle; see S2 Fig) [15, 25, 26], which mimics the AFM-based force measurements *in vitro*. In this approach, the mechanical loading of a biological particle is performed computationally (Methods and Models section) using MD simulations with experimental conditions of dynamic force application *f*(*t*) = *r*_*f*_ *t*. The significant computational acceleration available on Graphics Processing Units (GPUs) enables us to apply the experimentally relevant force-loading rates *r*_*f*_ = *κν*_*f*_ (*κ* is the cantilever spring constant), which correspond to the cantilever base velocity *ν*_*f*_ = 0.1–1.0 *μ*m/s. Structural transitions can be resolved by examining the coordinates of amino acid residues, and biomechanical characteristics can be accessed through analysis of the energy output.

Our *in silico* experiment provides the complete high resolution simulation view of particle deformation and collapse described below, where the choice of simulation conditions is entirely under the control of the investigator. The full control over the system during the nanomanipulations *in silico* can be used to study deformation at different specific symmetry points on the particle surface as well as the particle-indenter contact area dependence, and to relate the force and energy values recorded at any point in the simulation to the specific details observed in the particle’s structure. This type of precise high resolution control is not possible from nanoindentation carried out experimentally. Furthermore, when a sufficiently slow force loading is utilized our approach to nanoindentation *in silico* allows the investigator to follow the stochastic dynamics of mechanical deformation of a biological particle, which is microscopically reversible. In this regime of compressive force application, the rate of force increase is slower than the rate of system re-equilibration at each point along the deformation reaction path (quasi-equilibrium). For these reasons, we utilize our nanomanipulations *in silico* to guide the detailed modeling and interpretation of experimental results for the deformation dynamics of any biological nanoparticle being studied.

### Motivation for FNS model from MD simulation data

**Fig 1:**
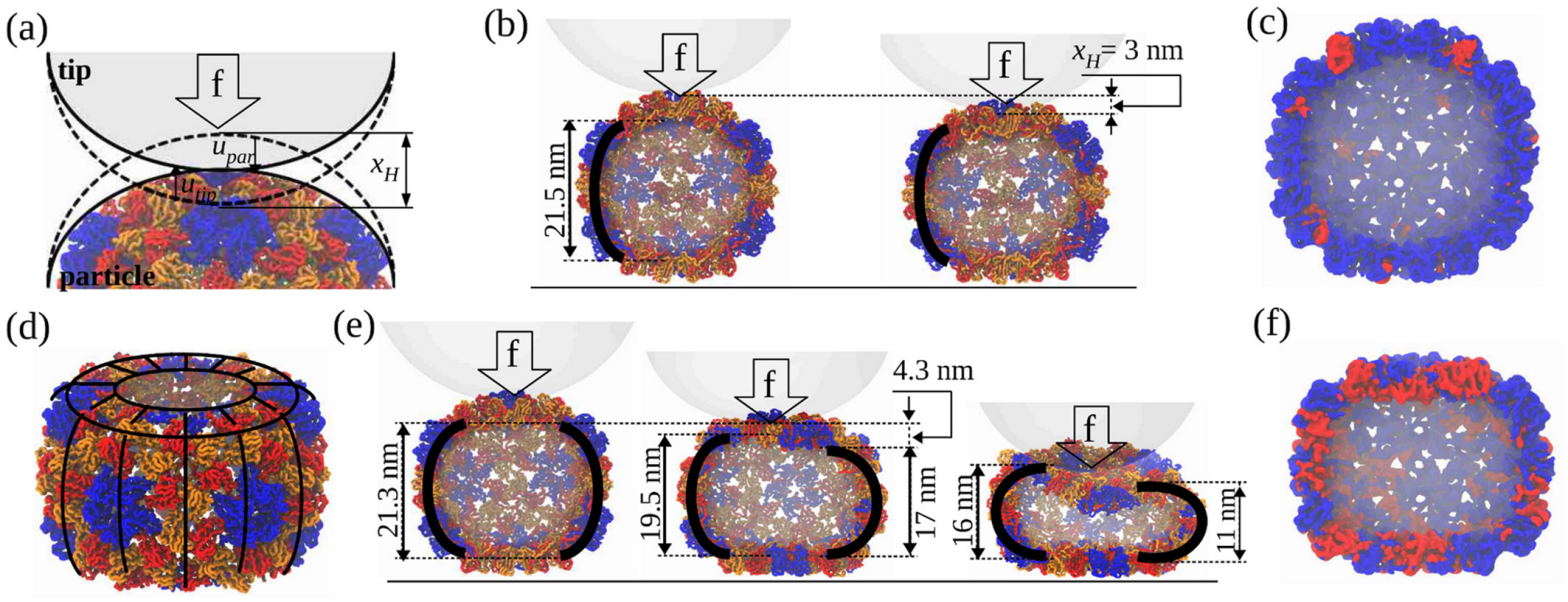
Types of mechanical excitations exemplified using the CCMV shell. (a)-(c) Hertzian deformation *x*_*H*_ with normal displacements *u*_*tip*_ and *u*_*par*_ (scheme on (a)) under the influence of force (vertical arrow). Dashed contour lines show the tip and particle in their undeformed states. Structures in (b)—the native (left) and partially deformed (right) states show an amplitude of *x*_*H*_ ≈ 3 nm. (c) CCMV shell profile showing parts of the structure with high potential energy (>3 kcal/mol per residue; red) and low potential energy (blue). (d)-(f) Bending deformation. The side portion of the structure (barrel) is partitioned into curved beams (top-side view on (d)). Structures in (e)—the partially deformed (left) and pre-collapse (middle and right) states reveal the amplitude of *x*_*b*_ ≈ 4.3 nm. (f) CCMV shell profile under Hertzian and bending deformations showing the potential energy distribution.

In this section, we will utilize MD simulation data to motivate the FNS model. Rigorous analyses of the structures and energy outputs from MD simulations of mechanical deformation of viruses [15] and a microtubule [26] showed that the mechanical response of biological nanoparticles, subject to a uniaxial deformation, can be divided into Hertzian and bending contributions (see Fig 1).

#### Hertzian and bending deformations

**Fig 2:**
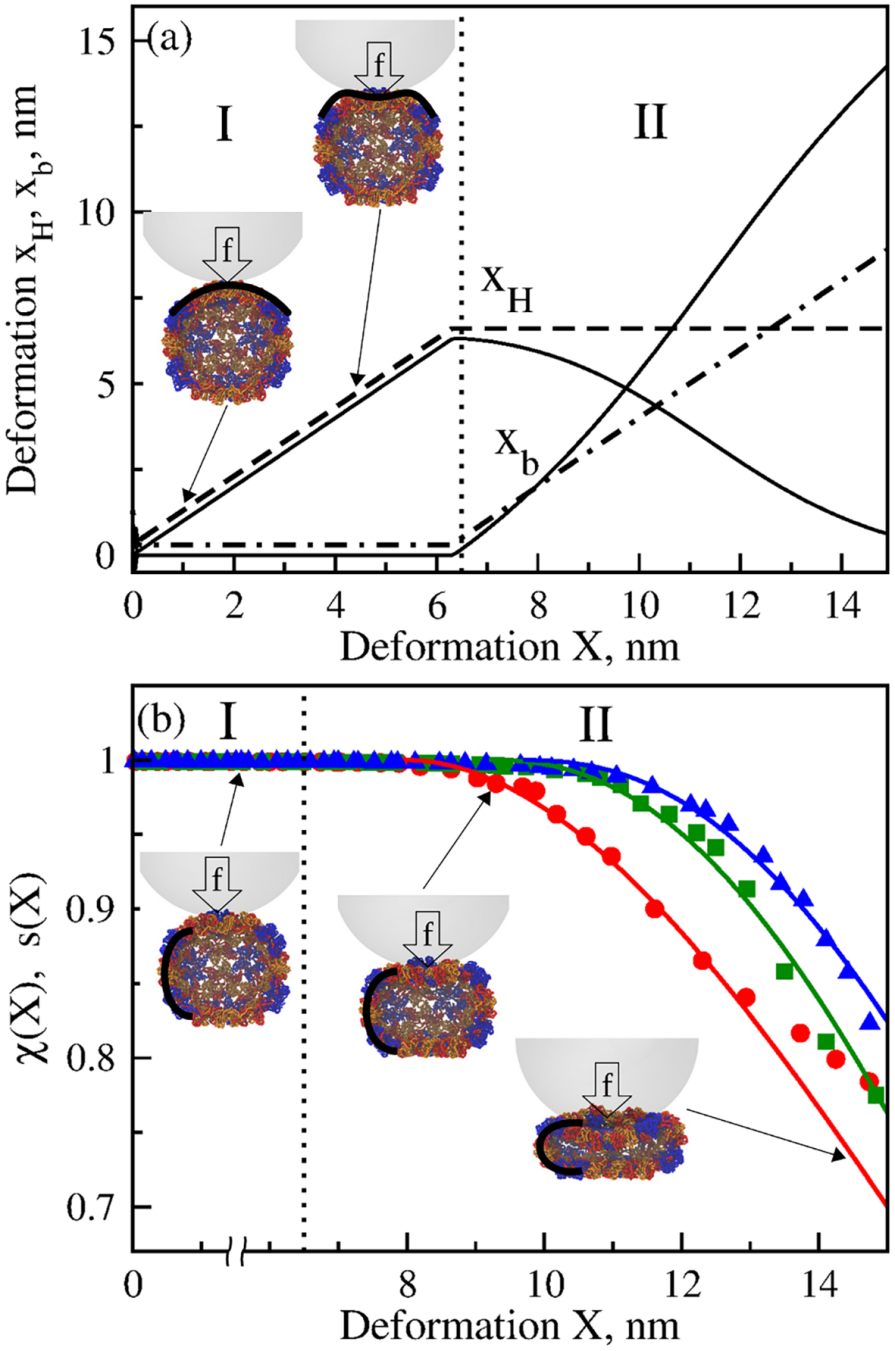
Dynamic evolution of mechanical degrees of freedom and survival probability for CCMV shell. Panel (a) exemplifies the dynamics of Hertzian deformation *x*_*H*_ and beam-bending deformation *x*_*b*_ vs. *X* in the Hertzian regime I and in the transition regime II. Model calculations are performed using parameter values obtained from the fit of theoretical *FX*-curves to the simulated average *FX*-spectra for CCMV nanoindentation along the 2-fold symmetry axis (Table 1). The solid curves correspond to the exact method of parameter estimation; the dashed and dashed-dotted curves are for the (piece-wise) approximate method of estimation. Snapshots exemplify the local flattening of CCMV structure under the tip for *X* = 1 nm and 5 nm deformation. Panel (b) displays the results of overlap function *χ*-based estimation of the survival probability *s*(*X*) from simulations of CCMV nanoindentation (*ν*_*f*_ = 1.0 *μ*m/s, *R*_*tip*_ = 20 nm, and *κ* = 0.05 N/m; S3a Fig) along the 2-fold (red), quasi-3-fold (blue), and quasi-2-fold symmetry axes (green). The theoretical profiles of *s*(*X*) (solid curves; see Eq (16)) are compared with the simulated profiles of *χ*(*X*) (data points; see Eq (15)). The model parameters are summarized in Table 1. The values of $x_{H}^{\max}$ are obtained using Lagrange multipliers and the approximate method of parameter estimation (see Discussion).

The Hertzian deformation accounts for the local compression of the protein layer (Fig 1a–1c, S1 Movie), which also results in an increase of the indenter-particle contact area. The bending deformations account for the evolution of the remaining portion of the particle structure (Fig 1d–1f, S1 Movie), which leads to the global collapse transition. These are distinctly different, independent excitations (both in the scale and direction of deformation), which become populated at different levels of mechanical stress. We separately analyzed the dynamics of Hertzian deformation (*x*_*H*_) and bending deformation (*x*_*b*_), and found that their dependence on *X* is similar to the dependence presented in Fig 2. We performed a careful analysis of the initial portion of a large number of experimental and simulated *FX* curves for the CCMV, TrV, and AdV particles (S3 and S4 Figs), and found that the weakly-nonlinear dependence of the indentation force (*F*) scales with deformation (*X*) as ∼*X* ^3/2^. We also analyzed the dependence of the size of contact area *a* on *X* and found, quite in agreement with the Hertz model, that *a* scales with *X* as ∼*X* ^1/2^ (not shown).

#### Parallel curved beams

To model bending deformations of the side-portion of the particle’s structure, we adopted the physical picture of coupled parallel beams undergoing mechanical deformations. First, all the biological particles studied have discrete structures. For example, the CCMV shell is formed by the structural integration of monomer protein assemblies termed pentamer and hexamer capsomers (S1 Fig); our previous studies revealed the uneven tension distribution in the pentamers and hexamers (Fig. S6 in the Supporting Information to Ref. [15]). Second, our simulations showed that mechanical loading is asymmetric, that is, at the onset of the transition to the collapsed state, certain structural elements of the “capsid barrel” are more loaded and, hence, yield sooner than others (S1 Movie). This can be gleaned from Fig 1e, which displays the results of a particular MD simulation trajectory (simulation run) of deformation and collapse of the CCMV shell. We see that due to fluctuations in the capsid structure, in this trajectory the “right beam” bends more than the “left beam”. This asymmetry grows with time and results in the beams undergoing sequential collapse transitions, which implies that some of the structural elements yield to force sooner than others. Therefore, microscopically various structural portions of the CCMV capsid collapse, but not all at the same time. Fig 1e also shows that the curvature change occurs in the side-portion of the shell where bending deformation develops along the beams’ contour length. These observations have led us to the physical picture of a collection of mechanically coupled beams.

#### Out-of-plane bending versus in-plane stretching

We focused on the out-of-plain bending of beams because our previous essential components analysis showed that this is the dominant mode, responsible for ∼85% of deformation dynamics (see Fig. S5 in Ref. [15]). However, the in-plane stretching modes can potentially contribute somewhat to deformation of protein domains forming the protein shell layer (e.g. in-plane deformation of pentamer/hexamer capsomers forming the virus capsid structure). To assess the relative importance of the “out-of-plane” bending mode as compared to the “in-plane” stretching mode of deformation, here we computed the distribution of the Cauchy stress tensor using the results of MD simulations for the CCMV particle. The Cauchy stress tensor per amino acid residue can be calculated using the formula:

$$\sigma_{i}^{\alpha \beta }=\frac{1}{2\Omega_{i}}\sum_{j}\frac{\partial U_{SOP}}{\partial r_{ij}}\frac{r_{ij}^{\alpha }r_{ij}^{\beta }}{r_{ij}},$$ (1)

where $\sigma_{i}^{\alpha \beta }$ is the Cauchy stress tensor for the *i*-th amino acid, *U*_*SOP*_ is the potential energy of the biological particle given by Eq. S1 (S1 Text), *r*_*ij*_ is the distance between the *i*-th and *j*-th particles, *α* and *β* denote coordinates *x*, *y* and *z* [27], $\Omega_{i}=4\pi /3a_{i}^{3}$ is the average volume of the *i*-th amino acid, and *a*_*i*_ is the average size of the *i*-th amino acid [28]. The normal stress component, corresponding to the “out-of-plane” bending, was calculated using the formula:

$$\sigma_{i}^{n}=\sum_{\alpha \beta }\sigma_{i}^{\alpha \beta }n^{\alpha }n^{\beta },$$ (2)

where **n** is the normal vector of indentation. The shear (tangential) stress component, corresponding to the “in-plane” stretching, was calculated using the formula:

$$\sigma_{i}^{\tau }=\sqrt{\sum_{\alpha \beta \gamma }\sigma_{i}^{\alpha \beta }\sigma_{i}^{\alpha \gamma }n^{\beta }n^{\gamma }-{\left(\sigma_{i}^{n}\right)}^{2}}$$ (3)

**Fig 3:**
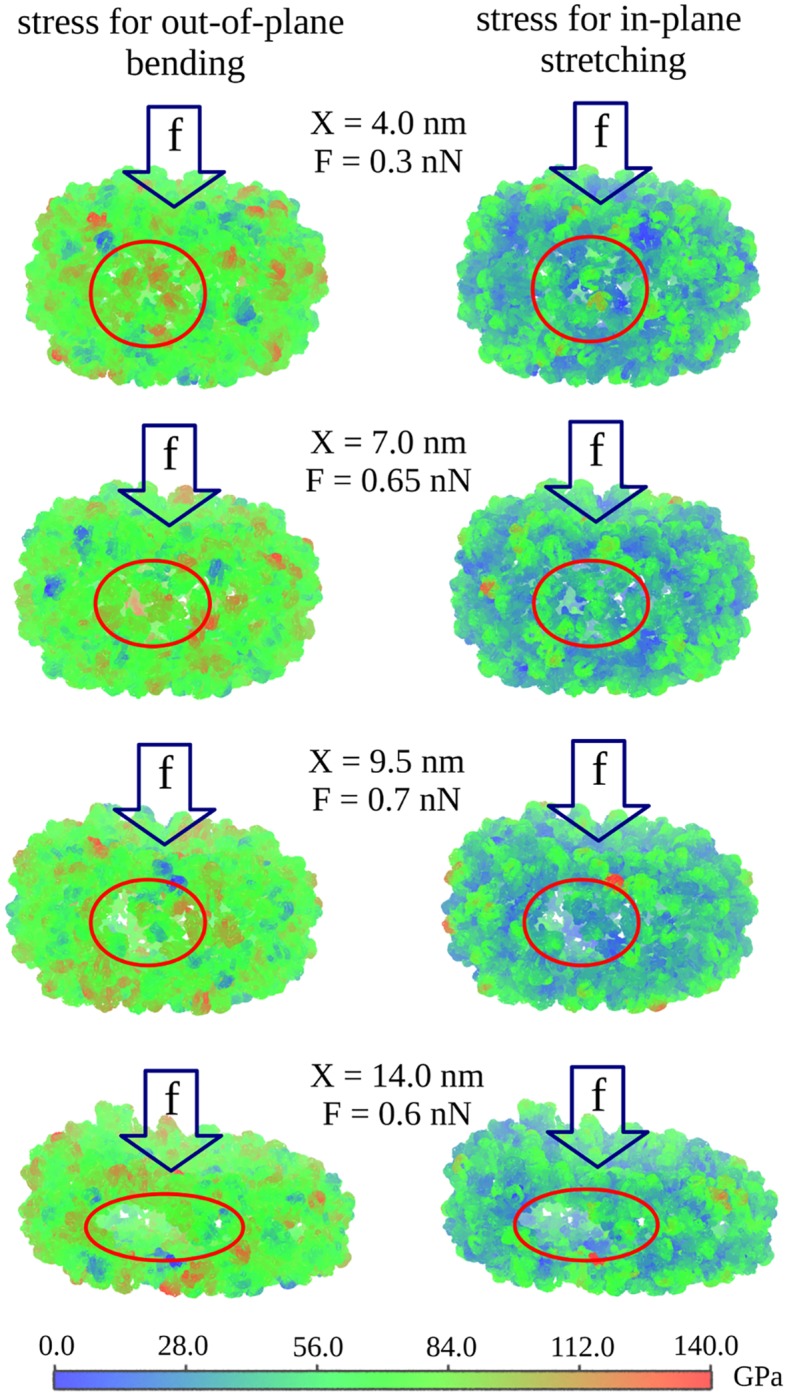
Stress distribution on CCMV shell surface. Map of the Cauchy stress tensor projections along the direction of out-of-plane bending deformation (left) and tangential in-plane stretching (right) for different deformation *X* of the CCMV shell and corresponding indentation force *F* (indentation along the 2-fold symmetry axis with *R*_*tip*_ = 20 nm and *ν*_*f*_ = 1.0 *μ*m/s). For each amino acid residue (*C*_*α*_-particle), the stress components are averaged over amino acids within a sphere of radius *R*_*C*_ = 15 Å (color denotation is presented in the graph). Also shown are formation and subsequent evolution of microscopic cracks in the side portion (particle barrel) of CCMV structure (shown in red circle/ellipse).

The results of calculation of the normal and shear stress components displayed in Fig 3 clearly show that the contribution to deformation dynamics from the “in-plane” stretching is small compared to that from the “out-of-plane” deformation. For this reason, the FNS model accounts only for the “out-of-plane” motions, which are far more significant to deformation dynamics than the “in-plane” motions.

### Identification of mechanical degrees of freedom for FNS model

In this section, we exemplify the FNS model in terms of the mechanical degrees of freedom that we identify to be most relevant to the uniaxial type of deformation. In dynamic force-ramp *f*(*t*) = *κν*_*f*_ *t*, an indenter (cantilever tip) compresses a particle (Fig 1 and S2 Fig), thus creating a physical contact between them. The force loads the particle mechanically over time *t* with the force-loading rate *κν*_*f*_ (*κ* and *ν*_*f*_ are the respective cantilever spring constant and velocity). For small force, the mechanical energy is localized to the particle surface under the tip, and the tip and particle undergo normal displacements *u*_*tip*_ and *u*_*par*_, corresponding to the deformation *x*_*H*_ = *u*_*tip*_+*u*_*par*_. Since *u*_*tip*_≪*u*_*par*_, *x*_*H*_ = *u*_*par*_. The force gradually loads the particle, stressing the side portions of the structure undergoing bending deformations *x*_*b*_ (Fig 1). Force-ramp conditions project the complex dynamics of the particle deformation in the direction perpendicular to the particle surface. During nanomanipulations *in vitro* and *in silico*, the deformation force *F*, the mechanical response of the particle, is measured as a function of the total deformation *X* = *x*_*H*_+*x*_*b*_ (reaction coordinate). Therefore, we focus here on the computation of the force-deformation (or *FX*) lineshape. We quantified *x*_*H*_ and *x*_*b*_ directly using the simulation output for the CCMV particle and found these to be independent, small-amplitude deformations. For example, the maximum values of *x*_*H*_ and *x*_*b*_ for the CCMV shell are 3 nm and 4.3 nm, respectively (Fig 1b and 1e).

Analysis of the experimental *FX*-spectra and structure snapshots from the MD simulations showed that the Hertz model [29, 30] properly accounts for the force *F*_*H*_ due to the observed local curvature change of the particle under the tip (Fig 1a),

$$F_{H}\left(x_{H}\right)=\frac{1}{D_{H}}\sqrt{\frac{R_{par}R_{tip}}{R_{par}+R_{tip}}}\cdot x_{H}^{3/2}$$ (4)

where *R*_*par*_ and *R*_*tip*_ are the radii of the particle and the tip, respectively. The term *D*_*H*_ is given by

$$D_{H}=\frac{3}{4}\left(\frac{1-\sigma_{H}^{2}}{E_{H}}+\frac{1-\sigma_{tip}^{2}}{E_{tip}}\right)$$ (5)

where *E*_*H*_ and *E*_*tip*_ are the Young’s moduli and *σ*_*H*_ and *σ*_*tip*_ are the Poisson’s ratios for the particle and the tip, respectively. Since *E*_*tip*_≫*E*_*H*_, $D_{H}=0.75\left(1-\sigma_{H}^{2}\right)/E_{H}$.

To describe the bending deformations *F*_*b*_(*x*_*b*_), we discretize the side portion of the particle structure (barrel) into curved vertical beams of length *L* (Fig 1d). The results of comparison of the out-of-plane bending and the in-plane stretching modes of deformation (Fig 3) showed that the effect of in-plane stretching on the total particle deformation is indeed negligible (see previous section). Hence, we can safely assume that the length of vertical beams *L* does not change with total deformation *X*. In view of the observations described above, our discretization of the particle barrel into curved vertical beams is fully justified. For a spherical particle of thickness *r*, the total number of beams is *N* = 2π*R*_*par*_/*r*. For small beam deformation *x*_*b*_ (Fig 1), the potential energy change is given by the integral *E*_*b*_ *I*/2∫_*L*_(*κ*(*x*_*b*_, *l*)−*κ*_0_)^2^ *dl* [29, 31], where *κ*_0_ and *κ*(*x*_*b*_, *l*) are the initial and instantaneous beam curvatures (0≤*l*≤*R* *_par_*−*x*_*b*_/2) and *E*_*b*_ *I* is its flexural rigidity, given by the product of the Young’s modulus for bending *E*_*b*_ and the moment of inertia *I*. With the beam shape function

$$q\left(x_{b},l\right)=\left(R_{par}+\frac{x_{b}}{2}\right)\sqrt{1-\frac{l^{2}}{{\left(R_{par}-x_{b}/2\right)}^{2}}}$$ (6)

the curvature is given by

$$\kappa \left(x_{b},l\right)=\frac{q^{\prime \prime }\left(x_{b},l\right)}{{\left(1+{\left(q^{\prime }\left(x_{b},l\right)\right)}^{2}\right)}^{3/2}}$$ (7)

where *q*′ and *q*″ are the first and second derivatives of *q* with respect to *l*. By performing the integration we obtain the expression for the bending energy, which upon differentiation with respect to *x*_*b*_, gives the bending force. Expanding the resulting expression in Taylor series in powers of *x*_*b*_ and retaining the linear term in the expansion, we obtain:

$$f_{b}\left(x_{b}\right)≅\frac{9E_{b}I\pi }{8R_{par}^{3}}\cdot x_{b}$$ (8)

**Fig 4:**
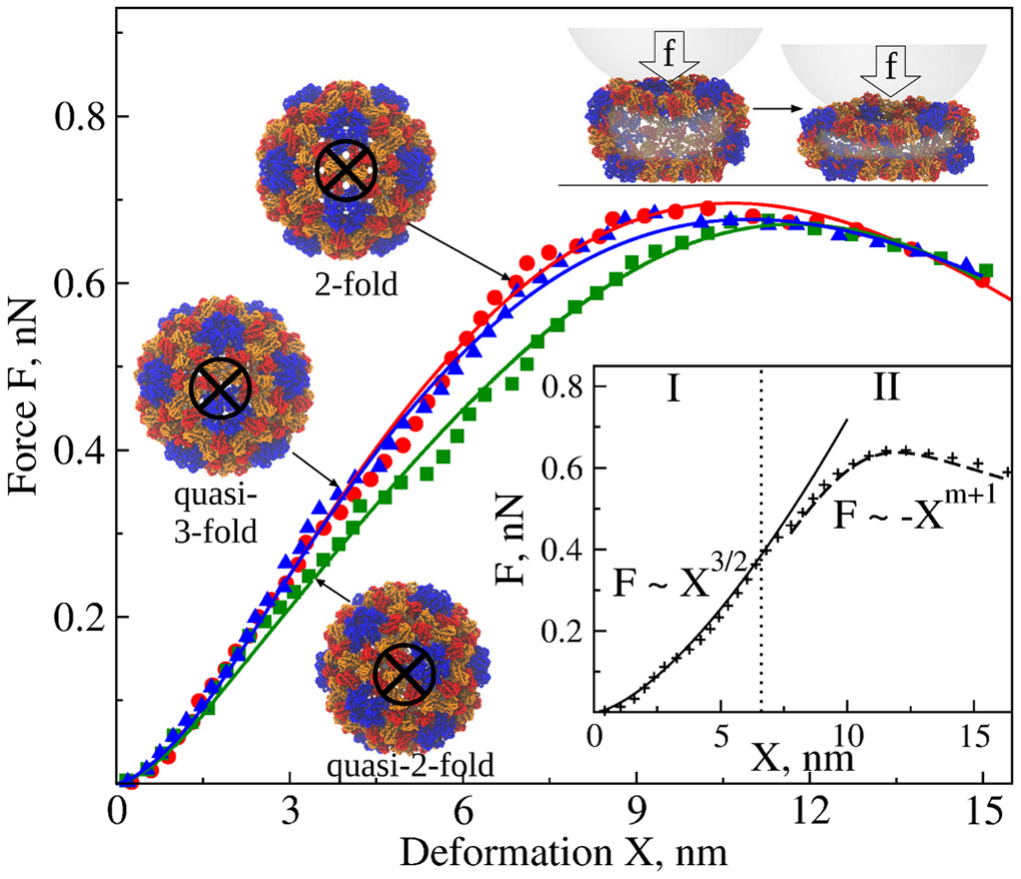
Compressive force-induced deformation of CCMV shell *in silico*. The average simulated *FX*-spectra (data points), obtained from nanoindentations *in silico* (*ν*_*f*_ = 1.0 *μ*m/s, *R*_*tip*_ = 20 nm, and *κ* = 0.05 N/m; S3a Fig) along the 2-fold (red), quasi-3-fold (blue), and quasi-2-fold (green) symmetry axes, are compared with the theoretical *FX*-curves obtained using the FNS model (solid lines). Snapshots on the left show the native CCMV structure in the intact state; the circled bolded X shows the locations of force application. We used MD simulations accelerated on GPUs [24, 25] and nanoindentations *in silico* to generate the average *FX*-spectra for the CCMV capsid (see also S1, S2, and S6 Figs). Computer simulation data are taken from Ref. [15]. Structures above the force maxima depict the capsid transitioning from the state right before the collapse (left) to the collapsed state (right). The *inset* shows a schematic for piece-wise spectrum modeling: in regime I, *X*≈*x*_*H*_, and *F*(*X*)≈*F*_*H*_; in regime II, *X* = *x*_*H*_+*x*_*b*_ and *F*(*X*) = *F*_*H*_+*F*_*b*_.

Combining the contributions from all *N* coupled elements (beams) and adding Eqs (4) and (8) together, we obtain the deformation force $\tilde{F}\left(x_{H},x_{b}\right)=k_{H}x_{H}^{3/2}+Nk_{b}x_{b}$, where *k*_*H*_ = (*R*_*par*_ *R*_*tip*_/(*R*_*par*_+*R*_*tip*_))^1/2^/*D*_*H*_ is the “Hertzian spring constant” and $k_{b}=9E_{b}I\pi /\left(8R_{par}^{3}\right)$ is the beam spring constant. In agreement with *in silico* indentations of CCMV shell (Figs 2 and 4) and recent experiments on thick-shelled particles [11], $\tilde{F}$ predicts that the initial portion of *FX*-curves is weakly nonlinear, but fails to capture the force drop (Fig 4) because the theory lacks a description of structural damage (see next section).

### Fluctuating Nonlinear Spring model

As we pointed out, owing to the discrete arrangements of capsomers forming the CCMV shell, structural elements fail but not all at the same time. To reflect the discrete nature of microscopic transitions, we represent a particle by a collection of *N* identical coupled elements (beams) interacting with an indenter through a Hertzian cushion (Fig 1). Each *i*-th beam undergoes the elastic deformation *x*_*bi*_ = *x*_*b*_ with the spring constant *k*_*b*_ until it fails mechanically when the load on the beam reaches some critical value $f_{bi}^{*}$ (see snapshots in Fig 4). The spherical geometry of a virus particle dictates the parallel arrangement with the spring $K_{b}=\sum_{i=1}^{N}k_{bi}=Nk_{b}$. At any given time, there are *n* (or *N*−*n*) beams that have failed (or survived), and the actual bending force is given by

$$F_{b}\left(x_{b}\right)=k_{b}\left(N-n\right)x_{b}=K_{b}x_{b}\left(1-\frac{n}{N}\right)$$ (9)

We define the probability of damage *d* = *n*/*N* and survival *s* = (*N*−*n*)/*N* = 1−*d* of the collection of beams, which in the continuous limit are described by the probability density function (pdf) *g*(*F*_*b*_), i.e.

$$d\left(F_{b}\right)=Prob\left(F_{b}\right)=\int_{0}^{F_{b}}g\left(F_{b}^{\prime }\right)dF_{b}^{\prime }$$ (10)

and *s*(*F*_*b*_) = 1−*d*(*F*_*b*_) (*F*_*b*_ = *K*_*b*_ *x*_*b*_). With the structural damage accounted for, the deformation force becomes:

$$F\left(x_{H},x_{b}\right)=k_{H}x_{H}^{3/2}+K_{b}x_{b}s\left(x_{b}\right)$$ (11)

Our rationale for using the survival probability measure *s*(*x*_*b*_) is based on the in-depth analysis of structures from the MD simulations of mechanical deformation of the CCMV virus [15] and microtubule polymers [26]. Both systems clearly demonstrate that soft biological particles accumulate structural damage. Fig 3 shows formation of small cracks in the CCMV shell, whereas Fig 1e provides a global view of the extent of structural damage in the CCMV particle, accumulated in the course of deformation.

The transition to the collapsed state occurs when all beams have failed, and so, the longest lasting beam determines the collapse onset at the critical deformation *X* ^*col*^ when tension exceeds the critical force *F* ^*col*^. Hence, the statistics of the maximum (extreme) force determines the beams’ failure. For these reasons, we used the two-parameter Weibull distribution [32]

$$\begin{array}{c} s\left(x_{b}\right)=\exp\left[-{\left(\frac{F_{b}}{F_{b}^{*}}\right)}^{m}\right]=\exp\left[-{\left(\frac{K_{b}x_{b}}{F_{b}^{*}}\right)}^{m}\right] \end{array}$$ (12)

with the cooperativity parameter *m*, and the scale parameter $F_{b}^{*}$. The meaning of $F_{b}^{*}$ can be understood by using the condition of maximum force, *dF*_*b*_/*dx*_*b*_ = 0, from which we obtain: $F_{b}^{*}=K_{b}x_{b}^{*}\sqrt[{m}]{m}$ where $x_{b}^{*}$ is the critical beam deformation. By substituting $F_{b}^{*}$ into the expression for *F*_*b*_(*x*_*b*_), we obtain the bending force threshold

$$\begin{array}{c} F_{b}^{col}=\frac{F_{b}^{*}}{\sqrt[{m}]{em}}=\frac{K_{b}x_{b}^{*}}{\sqrt[{m}]{e}} \end{array}$$ (13)

Finally, by substituting Eq (12) for *s*(*x*_*b*_) in Eq (11), we obtain one of the main results of the paper:

$$F\left(x_{H},x_{b}\right)=k_{H}x_{H}^{3/2}+K_{b}x_{b}\exp\left[-{\left(\frac{K_{b}x_{b}}{F_{b}^{*}}\right)}^{m}\right]$$ (14)

**Fig 5:**
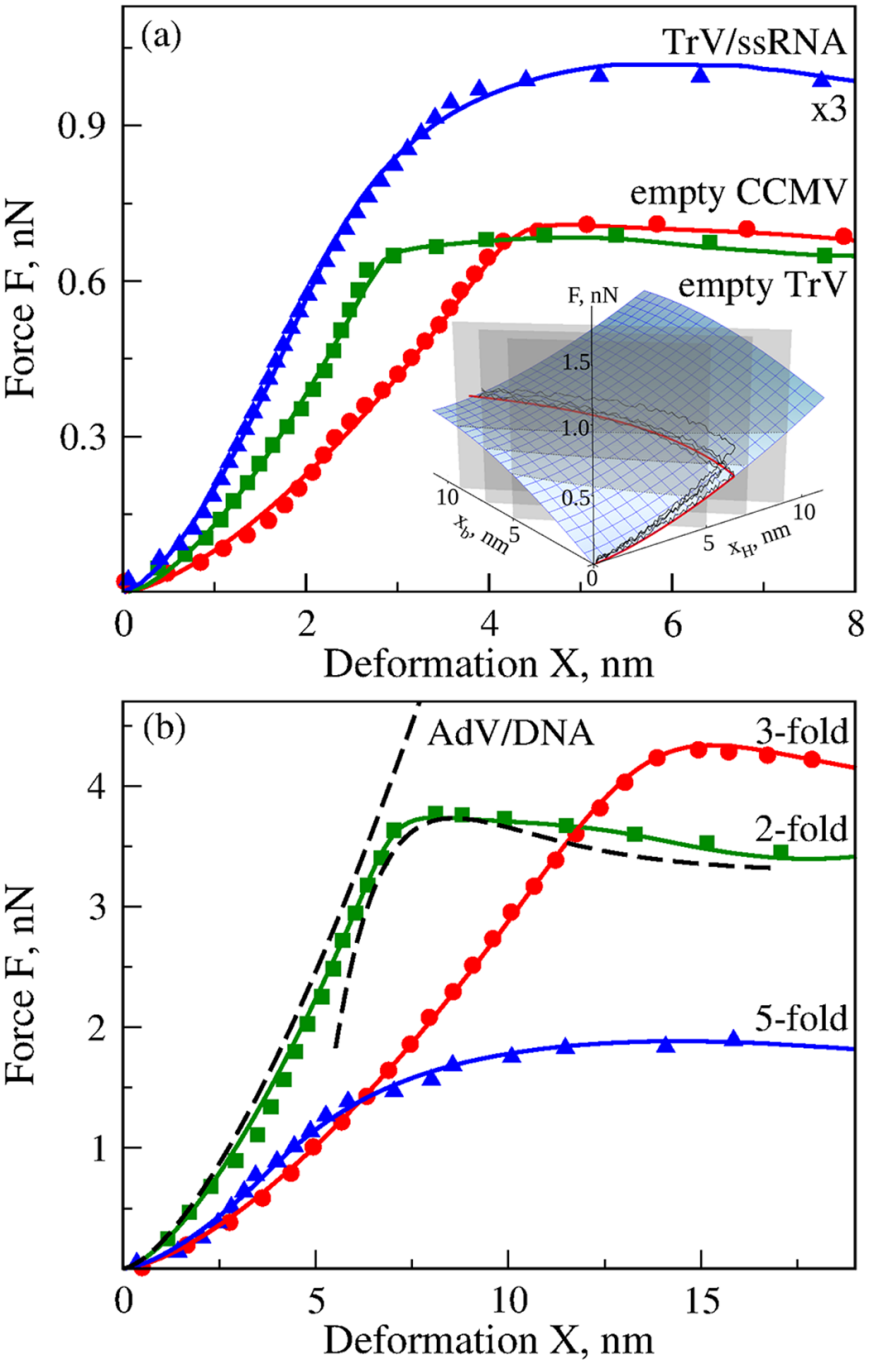
AFM-based compressive force-induced deformation of biological particles. Shown are the experimental results for empty CCMV shell, TrV shell and TrV particle with ssRNA (panel (a)), and AdV particle with dsDNA (panel (b)); see also S3b and S4a–S4c Figs. Experimental data are from Refs. [8, 12]. Please, see these publications for exact experimental procedures and results. The average experimental spectra (data points) are compared with (solid) theoretical *FX*-curves obtained using the FNS model. In panel (a), the *inset* shows the 2D-surface *F*(*x*_*H*_, *x*_*b*_) (Eq (14)) constructed using the model parameters from the fit of theoretical *FX*-curves to simulated *FX*-spectra obtained for CCMV indentation *in silico* along the 2-fold symmetry axis (Fig 4; Table 1). The red curve on the surface *F*(*x*_*H*_, *x*_*b*_) represents the equilibrium average path with the points formed by the intersection of *F*(*x*_*H*_, *x*_*b*_) surface with line *x*_*b*_ = *X*−*x*_*H*_ (shown using gray vertical plane for *X* = 5, 7 and 9 nm). The noisy black curves are particular realizations of the stochastic *FX*-path (i.e. individual *FX*-spectra). In panel (b), the dashed curves show a schematic for piece-wise modeling of the average experimental *FX*-spectrum for AdV particle deformation along the 2-fold symmetry.

The Fluctuating Nonlinear Spring (FNS) model describes the nonlinear deformation as a superposition of the weakly nonlinear deformation (Hertzian cushion) and the elastic deformation (particle barrel; Fig 1) of varying stiffness that is gradually degraded with *X*. Consequently, Eq (14) shows that the uniaxial deformation and structural collapse of a biological particle can be represented by the mechanical evolution of a fluctuating weakly nonlinear spring. This behavior led us to propose the name of the model. The beams’ bending starts as elastic (*Nk*_*b*_), but becomes increasingly more stochastic near the collapse transition, thus explaining the variability of *F* ^*col*^ and *X* ^*col*^ in the experimental and simulated *FX*-spectra (see Figs 4 and 5; see also S3 and S4 Figs).

## Discussion

### Application of FNS model

The FNS model can be understood by adopting a picture of “a particle as barrel” under the Hertzian cushion. Then, Eq (14) can be viewed as the complex mechanical response function to describe a biological particle whose stiffness is degraded exponentially with *x*_*b*_ as $K_{b}\exp[-{\left(K_{b}x_{b}/F_{b}^{*}\right)}^{m}]$. The latter quantity can be taken as the “effective stiffness” as compared to the native state stiffness of the particle *K*_*b*_. If we multiply the number of beams *N* = 2π*R*_*par*_/*r* by *k*_*b*_ (given by the prefactor in Eq (8)), we obtain $K_{b}=Nk_{b}=9E_{b}I\pi^{2}/4R_{par}^{2}r$, which carries information about the “particle barrel” (no information about beams). Also, in Eq (14), the shape parameter *m* can be interpreted as a cooperativity parameter that takes into account dynamic coupling among the beams. When *m* = 1 the beams are independent, which corresponds to the exponential distribution for the survival probability $s\left(x_{b}\right)=\exp[-K_{b}x_{b}/F_{b}^{*}]$. When *m* ≠ 1, the structural elements behave cooperatively to withstand the stress.

In the FNS model, the main quantity *F* is a bivariate function (Eq (14)), but in the experiment *F* is measured as a function of the sum *X* = *x*_*H*_+*x*_*b*_. To resolve *x*_*H*_ and *x*_*b*_ for each value of *X* we use the following considerations. A particular realization of the deformation process (*FX*-trajectory) is a stochastic path on the 2D surface *F*(*x*_*H*_, *x*_*b*_) displayed in Fig 5. For slow loading, when the particle structure equilibrates on a timescale faster than the rate of force change, the dominant path is the equilibrium deformation path. Utilizing slow cantilever velocities (*ν*_*f*_ = 0.06–1.0 *μ*m/s) allows us to use this quasi-equilibrium argument. Importantly, our recent study of the dynamics of deformation and collapse of microtubule polymers [26] show that *in silico* indentation experiments reported here are carried out under near-equilibrium conditions of compressive force application. Then, the equilibrium force can be determined from the requirement that the deformation force (and deformation energy) attains the minimum. Hence, finding the minimum force for each value of *X* is equivalent to finding *x*_*H*_ and *x*_*b*_, which minimize *F*(*x*_*H*_, *x*_*b*_) subject to the constraint, *X* = *x*_*H*_+*x* *_b_*. This can be solved using the method of Lagrange multipliers summarized in the S2 Text.

The average simulated spectra for the CCMV particle are compared with the theoretical curves in Fig 4 (simulated spectra for CCMV are accumulated in S3a Fig). To find the best fit, we employed two methods. The exact method is based on Eq (14) and uses Lagrange multipliers to find *x*_*H*_ and *x*_*b*_ subject to the constraint *X* = *x*_*H*_+*x* *_b_*. This approach can be used to model the average force-deformation spectra. The application of this method to describing the experimental or simulated force-deformation spectra requires solving the nonlinear equation for beam-bending deformation *x*_*b*_: $a_{1}x_{b}^{4m}+a_{2}x_{b}^{3m}+a_{3}x_{b}^{2m}+a_{4}x_{b}^{m}+a_{5}x_{b}+a_{6}=0$, where $a_{1}=m^{2}K_{b}^{2}{\left(K_{b}/F_{b}^{*}\right)}^{4m}$, $a_{2}=-2m\left(1+m\right)K_{b}^{2}{\left(K_{b}/F_{b}^{*}\right)}^{3m}$, $a_{3}=\left(1+4m+m^{2}\right)K_{b}^{2}{\left(K_{b}/F_{b}^{*}\right)}^{2m}$, $a_{4}=-2\left(1+m\right)K_{b}^{2}{\left(K_{b}/F_{b}^{*}\right)}^{m}$, $a_{5}=9/4k_{H}^{2}$, and $a_{6}=K_{b}^{2}-9/4k_{H}^{2}X$ are constant coefficients. Then, *x*_*H*_ is obtained as *x*_*H*_ = *X*−*x*_*b*_. In the piece-wise approximate method (see *inset* in Fig 4), a spectrum is divided into the Hertzian-deformation-dominated initial regime I: *X*≈*x*_*H*_ (*x*_*b*_ ≈ 0) and *F*≈*F*_*H*_ = *k*_*H*_ *X* ^3/2^; and the transition regime II (corresponding to the non-monotonic part of the *FX*-curve): *X*≈*x*_*b*_ and *F*≈*F*_*b*_ = *K*_*b*_ *Xs*(*X*). We calculate *F*_*H*_ in regime I for $X\approx x_{H}<x_{H}^{max}$, where $x_{H}^{max}$ is obtained using Lagrange multipliers and setting *s*(*x*_*b*_) = 1. In regime II, we use $F\left(x_{H}^{max},x_{b}\right)=k_{H}{\left(x_{H}^{max}\right)}^{3/2}+K_{b}\left(x_{b}-x_{H}^{max}\right)\exp[-{\left(K_{b}\left(x_{b}-x_{H}^{max}\right)/F_{b}^{*}\right)}^{m}]$ for $X\approx x_{b}>x_{H}^{max}$. This method can be used to model individual *FX*-spectra (displayed in S3 and S4 Figs) in order to access the entire distributions of a particle’s mechanical and statistical characteristics and to probe the variability of these properties due to the intrinsically stochastic nature of mechanical deformation and collapse of biological particles.

### Mechanical properties of CCMV, AdV, and TrV particles

We applied the FNS model-based theory to describe *FX* curves for the CCMV particle. The agreement between the simulated force-deformation spectra and theoretical *FX*-curves for the CCMV particle is very good (Fig 4). The *FX*-spectra presented in Fig 4 also fully agree with the *FX*-spectra for the CCMV particle discussed extensively in our previous study [15] in terms of the critical force *F* ^*col*^, critical deformation *X* ^*col*^, and the slope *dF*/*dX*. Simulated *FX*-curves show smaller variability as compared to the experimental *FX*-spectra, because in experiments not only are 2-fold, 3-fold, and 5-fold icosahedral orientations probed, but also various intermediate orientations. Less sharp force peaks due to slower force decrease observed in simulations can be attributed to overstabilizing the inter-chain interactions and neglecting the hydrodynamic interactions in the SOP model of the CCMV shell (work in progress). The values of model parameters obtained using both methods of estimation of the contributions *x*_*H*_ and *x*_*b*_ are very close (Table 1). For all symmetry types, the Hertzian excitation is softer than the bending (*k*_*H*_ < *K*_*b*_), implying smaller Young’s modulus, *E*_*H*_ < *E*_*b*_, which is why the Hertzian degree of freedom is excited first (regime I; see Fig 1). After the Hertzian force reached the maximum $F_{H}^{max}=k_{H}{\left(x_{H}^{max}\right)}^{3/2}$ at $X\approx x_{H}=x_{H}^{max}$, a subsequent force increase excites the beam-bending degrees of freedom (regime II) and *x*_*H*_ (*x*_*b*_) decreases (increases); see Fig 2a. Hence, the physical properties of the particle are dynamic (rather than static) since the nature of its mechanical response changes with increasing *X* from Hertzian-type to beam-bending deformation. The gradual decrease in *x*_*H*_ is somewhat counter-intuitive as one expects that *x*_*H*_ (and *F*_*H*_) remains constant after it has reached the maximum $x_{H}^{max}$ (and $F_{H}^{max}$). This is because the actual stiffness of beams is not constant but is degraded with increasing *x*_*b*_ due to the consecutive beam failure events (and accumulated damage). Therefore, in the transition regime II, the beam-bending *x*_*b*_ increases not only due to the continuing mechanical loading, but also as a result of stress redistribution to intact beams.

**Table 1:** Deformation and collapse of biological particles—CCMV, TrV, and AdV. Accumulated are the Young’s moduli for Hertzian *E*_*H*_ and bending *E*_*b*_ deformations, the beam strength $F_{b}^{*}$ and the cooperativity parameter *m*. The first (second) entries correspond to the exact (approximate) methods of parameter estimation. The model predictions for *F* ^*col*^ are compared with the peak forces (in parenthesis) from the spectra (Figs 4 and 5). For TrV and AdV particles, the shell thickness was estimated as described in the S3 Text.

**System**	**E_H_, GPa**	**E_b_, GPa**	$F_{b}^{*}$, nN	**m**	**F^col^, nN**
CCMV (2-fold symmetry; *in silico*)	0.013/0.012	0.50/0.50	1.70/1.25	1.7/1.5	0.67/0.69 (0.68)
CCMV (quasi-2-fold symmetry; *in silico*)	0.011/0.011	0.37/0.35	1.50/1.25	1.4/1.6	0.58/0.64 (0.68)
CCMV (quasi-3-fold symmetry; *in silico*)	0.012/0.012	0.52/0.46	1.75/1.33	1.4/1.6	0.58/0.64 (0.68)
empty CCMV (average; *in vitro*)	0.019/0.023	0.85/0.81	1.90/1.00	1.2/1.3	0.56/0.78 (0.71)
empty TrV (average; *in vitro*)	0.030/0.036	0.94/0.81	1.90/1.1	1.1/1.2	0.70/1.02 (0.69)
full TrV (average; *in vitro*)	0.140/0.140	0.95/0.84	8.10/5.5	1.1/1.0	2.91/3.78 (3.00)
full AdV (2-fold symmetry; *in vitro*)	0.037/0.040	0.35/0.29	10.0/5.0	1.2/1.4	2.58/4.05 (3.80)
full AdV (3-fold symmetry; *in vitro*)	0.018/0.019	0.20/0.18	11.0/5.0	1.3/1.7	3.04/4.15 (4.30)
full AdV (5-fold symmetry; *in vitro*)	0.021/0.023	0.14/0.13	5.10/3.7	1.1/1.0	2.03/2.35 (1.90)

The FNS model also explains why the mechanical response of biological particles depends on the structure of the particle-indenter contact and the particle and indenter geometries [15]. The parameter obtained from the model for different symmetries show that the mechanical response of CCMV varies with the location of compressive force application (Table 1). As all virus shells reflect the discrete symmetry of their specific capsomer arrangements, these results imply that the mechanical properties of virus particles are local (i.e. location-specific) characteristics of their structure. Furthermore, we found in our previous studies of near-spherical virus particles [15] and cylinder-shaped microtubule polymers [26] that the deformation force depends on the indenter size. The FNS model fully accounts for this finding. In the FNS model, the information about the particle and indenter geometries is contained in the Hertzian spring constant *k*_*H*_. Hence, the model predicts that the geometric effects are important only in the initial Hertzian-deformation dominated regime (regime I; see Fig 4). Application of the FNS model to several nanoscale biological particles (CCMV, AdV, and TrV virus shells) revealed that all exhibit *m*>1, which means that the structural elements forming the side-portion of the biological particle structure are mechanically coupled. For example, for the CCMV particle we found that for all indentation locations, the range of values was 1.8<*m*<2.1 (Table 1). Therefore, positive cooperativity is exhibited by the side-portion of the particle’s structure (beams), regardless of the point of indentation. Interestingly, the beams do not just fail when $F>F_{b}^{*}$, but begin to fail under smaller force $F_{b}^{col}=F_{b}^{*}/\sqrt[{m}]{em}$. For example, for *m* ≈ 2, we obtain $F_{b}^{col}\approx 0.43F_{b}^{*}$.

The AFM-based measurements for the empty CCMV shell, empty TrV capsid, full TrV virion (with encapsulated ssRNA molecule) and full AdV virion (with encapsulated dsDNA) are presented in S3b and S4 Figs. Theoretical fits to the experimental average *FX*-curves shows that their deformations are well described by the FNS model (Fig 5). The obtained Young’s moduli for Hertzian deformation are uniformly smaller (∼10–100 MPa) than the Young’s moduli for bending deformation (Giga-Pascal range; Table 1). There are small variations in the model parameters for the AdV virion due to force application at locations with different symmetry axes. This correlates with our similar findings for the CCMV shell, implying that the symmetry of local arrangements of capsomer repeats at the point of indentation influences its mechanics [15]. The values of cooperativity parameter are found to be greater than unity (*m* > 1), representing positive cooperativity, for all the systems studied. Parameters for empty and ssRNA-loaded TrV capsids indicate that the difference in particle stiffness is largely due to an increase in the Young’s modulus for Hertzian deformation *E*_*H*_ = 0.03 GPa (empty TrV) vs 0.14 GPa (full TrV), which suggests that local indentations are resisted in ssRNA-filled particles. These results fit with the previously observed deformation of RNA-filled TrV into an oblate sphere to maximize the volume available to pack the genome [12]. Hence, confining the large ssRNA genome inside the small particle volume builds internal pressure resisting local indentation. This behavior is in agreement with the general property of bacterial and higher organism viruses that have evolved to achieve maximum nucleic acid packing into the available virion volume, often exhibiting significant internal pressures in the mature packaged state. It is known that genomic material is one of many factors that influence nanoparticles’ mechanics, as described, in one example, in our previous study of TrV [12]. In full accord with this notion, the FNS model predicts that the presence of the genome defines the stability and physical properties of native virus particles. The biochemical properties of the nanoparticle shell are defined by the intra- and intersubunit protein interactions, and these non-covalent interactions are fully reflected in the SOP-model and they show up in the simulated *FX*-curves.

Previously, the 3D Young’s modulus of the capsid material was estimated by investigators using a thin shell theory [1, 11, 12, 29]. This assumption is valid for some bacteriophage capsids, but is not so in the case of CCMV and TrV capsids where the shell thickness cannot be neglected with respect to the virion radius. The FNS model properly accounts for compression of the protein layer under the tip. In the FNS model, the beam-bending modulus (*E*_*b*_) is roughly equivalent to the 3D Young’s modulus in the thin shell theory. It is estimated at ∼0.85 GPa (experiment) and ∼0.4–0.5 GPa (simulations) for the empty CCMV capsid (Table 1). These are similar to yet larger than the values of 0.15–0.30 GPa obtained with thin shell theory [1, 11] and 0.28–0.36 GPa from finite-element analysis (∼0.25 GPa) [33], but they disagree with the estimates from several computer modeling studies (0.08–0.09 GPa) [22, 23]. In the modeling study based on spherical harmonics [23], multiple deformation modes have also been observed, corresponding to equilibrium deformations of the polar regions (tip-surface contact area in FNS model) and the side wall (beams in FNS model) of the shell. For the empty TrV capsid, we obtain *E*_*b*_ ≈ 0.9 GPa (Table 1) whereas the thin shell theory gives ∼0.5 GPa. The lower previous estimates of the 3D Young’s modulus result from attributing the softer Hertzian deformation mode to bending of the capsid shell in the thin shell theory. Indeed, for CCMV and TrV, the thin shell theory estimates of 0.15–0.30 GPa and 0.5 GPa are between the values of *E*_*H*_ = 0.02–0.03 GPa and *E*_*b*_ = 0.85–0.95 GPa from the FNS-model based modeling (Table 1).

### Structure-based interpretation of survival probability

One of the novel aspects of the FNS model is that it allows one to interpret the survival probability $s\left(x_{b}\right)=exp\left[-{\left(K_{b}x_{b}/F_{b}^{*}\right)}^{m}\right]$ (Eq (12)) using the concept of structural similarity quantified by the structure overlap function *χ*(*x*_*b*_). *In silico*, *s*(*x*_*b*_) can be directly accessed by calculating the structural similarity *χ* between a given (current) structure (corresponding to beam-bending deformation *x*_*b*_) and the native (reference) state, using the formula:

$$\chi \left(x_{b}\right)={\left(2M\left(M-1\right)\right)}^{-1}\sum \Theta (|r_{ij}\left(x_{b}\right)-r_{ij}\left(0\right)|-\beta r_{ij}\left(0\right))$$ (15)

In Eq (15), *M* is the total number of amino acid residues comprising the particle’s structure (system size), and in the Heaviside step function Θ(*x*), defined as Θ = 0 for *x*<0 and Θ = 1 for *x*≥0, *r*_*ij*_(*x*_*b*_) and *r*_*ij*_(0) are the distances between the *i*-th and *j*-th amino acids in the given and native structures, respectively (*β* = 0.2 is the tolerance for distance change).

Since Hertzian deformation is local, i.e. it is limited only to the protein domains in and around the indenter-particle contact area (see Fig 1a–1c), this type of deformation does not significantly affect the global particle structure, and so *χ*(*X*)≈*χ*(*x*_*b*_). Indeed, the *X*-dependent profiles of *χ* show that the structure overlap decreases at large values of *X* only when mechanical loading starts deforming the beams (Fig 2b). On the other hand, *s*(*x*_*b*_) decreases only after the Hertzian deformation has reached the maximum $x_{H}=x_{H}^{max}$. At this point, a subsequent increase in *X* loads the beams, resulting in the increase of *x*_*b*_ and decrease of *s*(*x*_*b*_) (Fig 2a). Hence, the dependence of *s* on *X* can be approximately described as

$$s\left(X\right)\approx s_{0}\Theta \left(x_{H}^{max}-X\right)+s\left(X-x_{H}^{max}\right)\Theta \left(X-x_{H}^{max}\right)$$ (16)

In Eq (16), *s*_0_ = 1 represents the initial values of *s*(*X*) in the Hertzian deformation regime I, and the second term on the right describes the dependence of *s*(*X*) in the beam-bending regime II (see Fig 2 and the *inset* to Fig 4). Because the structure overlap *χ* ranges from *χ* = 1 (identical structures) to *χ* = 0 (completely dissimilar structures), structural alterations and, hence, changes in *χ* can be translated to changes in *s*, i.e.

s(X)≈χ(X) (17)

Therefore, as Eq (17) implies, the survival probability *s*(*X*) can also be modeled using the structure data from nanoindentation simulations.

To confirm the above conclusion, we estimated *s*(*X*) using the structure output from *in silico* nanoindentations of the CCMV particle. The structure overlap *χ*-based estimation of *s*(*X*) (data points; Eq (17)) and theoretical profiles of *s*(*X*) (curves; Eq (16)) are directly compared in Fig 2b. The results of comparison fully confirm this conclusion, and also demonstrate that the survival probability *s*(*X*) has a well-defined interpretation in terms of the particle’s structure. Stated differently, this probability measure is not some intermediate variable used to formulate the theory, but rather, it is an important ingredient of the FNS model. Hence, in the theoretical framework of the FNS model, the survival probability *s*(*X*) provides a direct link between the dynamic structural changes observed in biological particles and the intrinsically stochastic nature of their deformation and transition to the collapsed state. In a sense, the FNS is also a structure-based model. In this regard, the structure overlap function *χ*(*X*) can be utilized in conjunction with the structure output from nanoindentations *in silico* to guide the modeling efforts in order to resolve *s*(*X*).

### FNS model predictions

The proof of a theory is in its predictive power. First, we used parameters of the FNS model (Table 1) to calculate the position *X* ^*col*^ and the amplitude of the force peak (force maximum) *F* ^*col*^ for the average *FX*-spectra (Figs 4 and 5), and to predict the critical force for collapse:

$$X^{col}=x_{H}^{*}+x_{b}^{*}\;\text{and}\;F^{col}=F_{H}\left(x_{H}^{*}\right)+F_{b}^{col}\left(x_{b}^{*}\right)=k_{H}{\left(x_{H}^{*}\right)}^{3/2}+\frac{K_{b}x_{b}^{*}}{\sqrt[{m}]{e}}$$ (18)

Remarkably, the obtained theoretical values of *F* ^*col*^ (Table 1) agree well with their counterparts extracted from the average *FX*-curves, which validates the model.

Second, individual *FX*-curves display large variability of critical deformations and critical forces (see S3b and S4 Figs). In the FNS model, this information is implicitly contained in the survival probability *s*(*x*_*b*_) and damage probability *d*(*x*_*b*_). The width of the transition region, in which *s*(*x*_*b*_) (*d*(*x*_*b*_)) decrease (increase) to zero (unity), defines the range of critical deformations Δ*X* ^*col*^. Upon rescaling, *K*_*b*_ *x*_*b*_→*F*_*b*_, *s*(*x*_*b*_) and *d*(*x*_*b*_) are transformed into the force probabilities *s*(*F*_*b*_) and *d*(*F*_*b*_), and the width of the transition region for *s*(*F*_*b*_) and *d*(*F*_*b*_) defines the range of critical forces Δ*F* ^*col*^. As an example, we estimated Δ*X* ^*col*^ by analyzing the transition range for the survival probability *s*(*X*) given by Eq (16). We used the FNS model parameters obtained for experimentally tested empty CCMV particle from Table 1 (Fig 5a; see also S3b Fig for individual *FX*-curves). The results of estimation of the transition range for the CCMV shell using *s*(*X*) are displayed in S5 Fig. We obtained Δ*X* ≈ 8.0 nm (shaded area in S5 Fig) which compares well with the experimental value Δ*X* ^*col*^ = 6 nm. The corresponding range of critical forces, Δ*F* = *K*_*b*_Δ*x*_*b*_ = *K*_*b*_Δ*X* = 0.24 nN/nm × 8 nm ≈ 1.9 nN, compares well with the experimental range Δ*F* ^*col*^ = 0.7 nN. Clearly, the experimental ranges for both Δ*X* ^*col*^ and Δ*F* ^*col*^ are shorter than the theoretical widths Δ*X* and Δ*F* due to a limited number of experimental measurements (7 runs; see S3b Fig).

We have demonstrated that the FNS model based theory: (i) correctly predicts the location of the force peaks *X* ^*col*^ and amplitude of peak forces *F* ^*col*^ extracted from the average *FX*-spectra, and (ii) describes the variability of critical deformations and critical forces around their average values (*X* ^*col*^ and *F* ^*col*^). About half of all known viruses possess icosahedral symmetry [34] and, therefore, here we focused on examples of virus particles with this symmetry. However, the model can be applied much more widely to characterize a range of biological nanoparticles, for which the *FX*-spectra are already available, including plant and animal viruses and bacteriophage, cellular nanocompartments, cytoskeletal polymers, etc. Although the FNS model is tailored to treat small deformations, it can be used to account for large deformations as well. This would require the extension of Eq (8) to include the higher order terms in *x*_*b*_. Also, the Hertz model could be improved to account for the non-local deformations.

The FNS model can be used to interpret the *FX*-curves for biological particles of different regular geometries, including cylindrical or ellipsoidal shapes, as long as the particles are subjected to a uniaxial compressive force induced by a spherical-like indenter. Extension of the FNS model to other indenter geometries is also possible. In this paper, however, we used the “Hertzian spring constant” *k*_*H*_ to treat the sphere-sphere interaction, because our goal was to explore the mechanical deformation of virus particles, which are nearly spherically-shaped, and because the cantilever tips used in AFM experiments can be approximated by a sphere. Also, when nanoindentation measurements are performed using a smaller tip compared to the size of the biological particle (which is a typical situation realized in AFM experiments), the tip-particle contact area is roughly circular. For these reasons, in this paper we treated the simplest case of near-circular contact area. We will discuss these geometry-related aspects of the FNS model in future work, including a more general case of elliptic particle-indenter contact area (manuscript in preparation).

### Conclusions

Living organisms have evolved with hierarchical supramolecular systems playing key roles in their biological functions. The dynamic properties of spontaneous assembly, disassembly, and self-repair exhibited by supramolecular assemblies explains their central importance. Prime examples of hierarchical supramolecular assemblies are the easily studied plant and animal viruses and bacteriophages. Although well studied, it remains a challenge to elucidate the structural origins of their unique physico-chemical properties as well as to resolve the specific mechanisms of their response to a wide variety of both biochemical molecules and external mechanical factors. In conjunction with single-molecule techniques, like AFM, dynamic force spectroscopy has become a nearly routine discovery tool for understanding the physical properties of intact biological particles [1]. However, the results of such experimentation remain difficult to interpret. In a number of our recent studies, we have developed an approach to nanoindentation *in silico* that involves multiscale modeling [15, 26]. The value in this novel approach is that it provides a toolbox for the computational interrogation of biomechanical properties that characterize large-size biological assemblies.

As a result of this recent progress in experimental and computational studies on forced indentation of biological nanoparticles, there is a growing need for a simple theoretical approach to quantitatively describe force-deformation curves. We developed the analytically tractable FNS model which uniquely combines the elements of continuum mechanics and statistics of extremes to accurately describe the uniaxial mechanical deformation and structural collapse (beyond buckling) in biological nanoparticles. The FNS model is based on a clear microscopic picture resulting from the multiscale modeling efforts, which involve direct atomistic and coarse-grained simulations of virus particles. However, it is important to note an application of the FNS model does not require the results of MD simulations as an input, and, hence, the FNS model can be applied widely to any regular geometry nanoparticle. To formulate the model, here we used: (i) virus deformation simulation data which agreed with experiment [15], (ii) data gathered at the nanometer scale (<1 nm), and (iii) experimentally relevant force-loading conditions. Due to the limited resolution of the AFM-based experimental technique, the only direct structural evidence is currently available from *in silico* experiments (Figs 1 and 2), which we have utilized in this paper to guide our modeling efforts.

We have demonstrated how the FNS theory can accurately model the deformation of viral nanoparticles, showing promising applications of this theory to describing the physics and mechanochemistry of a wide variety of both natural as well as synthetic nanoparticles. In the FNS theory, cooperativity parameter *m* may be of particular value. It allows for the direct comparison of energetic cooperativity magnitude differences between related nanoparticles that might be undergoing rationale design by investigators. As such, it could represent an important evaluation tool for structural alterations made with the aim to ultimately achieve optimal mechanical and energetic properties of natural and synthetic nanocompartments. In the case of natural viral nanoparticles, FNS theory may aid in revealing how mechanical properties correlate with local conformational dynamics of the capsid structure to contribute to crucial steps in the viral infectious cycle, such as receptor binding, genome uncoating and capsid maturation.

## Methods and Models

### Multiscale modeling approach

In our MD simulation studies, we employed multiscale modeling, which combines the simulations of atomic structural models [35] with amino acid residue (*C*_*α*_-atom) based Self Organized Polymer (SOP) model of biological particles [24, 25, 36, 37]. In this approach, we first use the all-atom Molecular Dynamics simulations of atomic structural models of a biological particle in question in implicit water using the Solvent Accessible Surface Area (SASA) model and Generalized Born (GB) model of implicit solvation. These equilibrium MD simulations are carried out in order to obtain an accurate parameterization of the SOP model, as described in the S4 Text. The atomic-level details that determine the type and number of residue-residue contacts between amino acids and their energies are then ported to the SOP model of the particle structure.

### Nanoindentation *in silico* method

In dynamic force measurements *in silico*, the cantilever base is represented by the virtual particle, connected to the spherical bead of radius *R*_*tip*_, mimicking the cantilever tip (indenter), by a harmonic spring (S2 Fig). The tip interacts with the particles via the repulsive Lennard-Jones potential:

$$U_{tip}=\sum_{i=1}^{N}\varepsilon_{tip}{\left(\frac{\sigma_{tip}}{|r_{i}-r_{tip}|-R_{tip}}\right)}^{6}$$ (19)

thereby producing an indentation on the particle’s outer surface. In Eq (19), *r*_*i*_ and *r*_*tip*_ are coordinates of the *i*-th particle and the center of the tip, respectively, *ε*_*tip*_ = 4.18 kJ/mol, and *σ*_*tip*_ = 1.0 Å are parameters of interaction, and the summation is performed over all the particles under the tip. For the cantilever tip (sphere in S2 Fig), we solve numerically the following Langevin equation of motion:

$$\eta \frac{dr_{tip}}{dt}=-\frac{\partial U_{tip}\left(r_{tip}\right)}{\partial r_{tip}}+\kappa \left(\left(r_{tip}^{0}-\nu_{f}t\right)-r_{tip}\right)$$ (20)

where $r_{tip}^{0}$ is the initial position of spherical tip center (*ν*_*f*_ is the cantilever base velocity; *κ* is the cantilever spring constant), and the friction coefficient *η* = 7.0 × 10^6^ pN ps/nm. To generate the dynamics of the biological particle of interest tested mechanically, we solve numerically Eqs. (S1)—(S5) for the particle (see S1 Text) and Eqs (19) and (20) for the indenter (spherical tip).

The cantilever base moving with constant velocity (*ν*_*f*_) (S2 Fig, S1 Movie) exerts (through the tip) the time-dependent force (force-ramp) f(t)=f(t)n in the direction **n** perpendicular to the particle outer surface. The force magnitude, *f*(*t*) = *r*_*f*_ *t*, exerted on the particle increases linearly in time *t* with the force-loading rate *r*_*f*_ = *κν*_*f*_. In the simulations of “forward indentation”, the cantilever base (and spherical tip) is moving towards the virus capsid. We control the piezo (cantilever base) displacement *Z*, and the cantilever tip position *X*, which defines the indentation depth (deformation). The resisting force of deformation *F* from the virus particle, which corresponds to the experimentally measured indentation force is calculated using the energy output from simulations. To prevent the capsid from rolling, we constrain the bottom portion of the particle by fixing selected *C*_*α*_-atoms contacting the substrate surface.

### AFM-based forced indentation experiments

The experimental *FZ*-spectra were obtained as described in our previous studies [8, 12, 38]. In short, hydrophobic glass slides were treated with an alkylsilane [2]. The viral samples were kept under liquid conditions at all times; all the experiments were performed at room temperature. Capsid solutions were incubated for ∼30 minutes on the hydrophobic glass slides prior to the indentation experiments. Olympus OMCL-RC800PSA rectangular, silicon nitride cantilevers (nominal tip radius <20 nm and spring constant of 0.05 N/m) were calibrated in air yielding a spring constant of *κ* = 0.0524±0.002 N/m. Viral imaging [39] and nanoindentation [1] were performed on a Nanotec Electronica AFM (Tres Cantos, Spain). For empty CCMV, *ν*_*f*_ = 0.06 *μ*m/s, *R*_*tip*_ = 20 nm, and *κ* = 0.05 N/m. For empty TrV, *ν*_*f*_ = 0.06 *μ*m/s, *R*_*tip*_ = 15 nm, and *κ* = 0.056 N/m. For TrV with ssRNA, *ν*_*f*_ = 0.06 *μ*m/s, *R*_*tip*_ = 15 nm, and *κ* = 0.1 N/m. For full AdV with dsDNA, *ν*_*f*_ = 0.055 *μ*m/s, *R*_*tip*_ = 15 nm, and *κ* = 0.0524 N/m. The indentation data were analyzed using a home-written Labview program (National Instruments) as described elsewhere [38]. To obtain force-deformation spectra (*FX*-curves) from the experimental output (*FZ*-curves), we employed the coordinate transformation from the *Z*-representation (*FZ*-curves) to the *X*-representation (*FX*-curves), i.e. *X* = *Z*−*F*/*κ* [40].

## Supporting Information

S1 Text Self Organized Polymer (SOP) model of a virus particle. (PDF)

S2 Text Method of Lagrange multipliers. (PDF)

S3 Text Estimation of the thickness of TrV and AdV with encapsulated genome. (PDF)

S4 Text SOP model parameterization for CCMV shell. (PDF)

S1 Fig The structure of the Cowpea Chlorotic Mottle Virus (CCMV) (PDB code: 1CWP). The side view of the CCMV shell is shown on the right. The protein domains forming pentamers are in blue, while the same protein domains in hexamers are in red and orange. The hexamers and pentamers, composed of six and five copies of the same protein chain (circled in the black ellipse), are displayed on the left. The CCMV capsid is a ∼2.8 nm thick shell with a ∼26 nm diameter.
(TIFF)

S2 Fig Schematic of the setup used in nanoindentations *in silico*. The biological particle (virus shell) is placed on the substrate. The cantilever base (virtual sphere) is moving in the direction perpendicular to the surface of the particle with the constant velocity *ν*_*f*_ (force-ramp), which creates a compressive force. The force is transmitted to the cantilever tip (sphere of radius *R*_*tip*_) through the harmonic spring with the spring constant *κ*. The force exerted on a particle *f*(*t*) = *r*_*f*_ *t* (large vertical arrow) ramps up linearly in magnitude with time with the force-loading rate *r*_*f*_ = *κν*_*f*_, which mechanically loads the particle. The mechanical response of the particle can be probed by profiling the deformation force (indentation force) *F* as a function of the cantilever base (piezo-) displacement *Z* (*FZ* curve) or as a function of the indentation depth *X* (*FX* curve).
(TIFF)

S3 Fig Nanoindentation of the empty CCMV particle *in silico* (a) and *in vitro* (b). Shown in different colors for clarity are the *FX* curves obtained using the cantilever tip velocity *ν*_*f*_ = 0.06 *μ*m/s (experiment) and *ν*_*f*_ = 1.0 *μ*m/s (simulations). In the AFM-based experiments and in simulations of nanoindentation of CCMV, we used the cantilever tip with radius *R*_*tip*_ = 20 nm and the spring constant *κ* = 0.05 N/m. In panel (a), structural snapshots from the left to the right, which correspond to the *FX* curve shown in blue, display the progress of forced deformation from the native un-deformed state (leftmost structure), to the partially deformed state (middle structures), and finally to the globally collapsed state (rightmost structure). In nanoindentation measurements *in silico* and *in vitro*, the cantilever tip indents the capsid in the direction perpendicular to the capsid outer surface (shown by a large vertical arrow). Simulation and experimental data are from Ref. [15] in the main text, please see this publication for exact experimental procedures and results.
(TIFF)

S4 Fig AFM-based nanoindentation of the empty TrV particle (a), full TrV particle (encapsulating the single-stranded RNA genome; (b)) and full AdV particle (encapsulating the DNA genome, (c)). Shown in different colors for clarity are the representative force-deformation spectra. The *FX* curves for the empty TrV particle were obtained using the cantilever tip velocity *ν*_*f*_ = 0.06 *μ*m/s, tip radius *R*_*tip*_ = 15 nm, and spring constant *κ* = 0.056 N/m. The *FX* curves for the full TrV particle were obtained using *ν*_*f*_ = 0.06 *μ*m/s, *R*_*tip*_ = 15 nm, and *κ* = 0.1 N/m. The *FX* curves for the full AdV particle were obtained using *ν*_*f*_ = 0.055 *μ*m/s, *R*_*tip*_ = 15 nm, and *κ* = 0.0524 N/m. Experimental data are from Refs. [8, 12] in the main text, please see these publications for exact experimental procedures and results.
(TIF)

S5 Fig Theoretical profile of the beam survival probability. Shown is the curve of *s*(*X*) obtained using Eq (16) with FNS model parameters for the empty CCMV particle tested experimentally (see Table 1 in the main text). The shaded area represents the width of the transition range Δ*X* ≈ 8.0 nm, which compares well with the experimental value of the same quantity Δ*X* ^*col*^ = 6 nm from statistical analysis of critical deformations (S3b Fig).
(TIF)

S6 Fig Graphical illustration of the coarse-graining procedure involved in construction of a SOP model of a polypeptide chain (see S1 Text). Panel (a) shows coarse-graining of the atomic structure of the protein subunit forming pentamers and capsomers of the CCMV shell (S1 Fig). Each amino acid residue is represented by a spherical bead of an appropriate radius with the coordinates of the *C*_*α*_-atom (black circles). The protein backbone is replaced by a collection of the *C*_*α*_-*C*_*α*_ covalent bonds with 3.8 Å bond distance. The potential energy function (see Eq. S1 in S1 Text) describes the interactions between amino acids stabilizing the native state of the protein chain, and the chain connectivity, elongation due to stretching, and self-avoidance. The coarse-graining procedure preserves the secondary structure: *α*-helices (pink), *β*-strands and sheets (blue), and random coil and turns (gray). Panel (b) shows the results of coarse-graining of a hexamer. Six identical copies of the same protein monomer (coarse-grained in (a)) form a *C*_*α*_-based model of the hexamer subunit. The hexamers and pentamers are combined to form a coarse-grained reconstruction of the full CCMV shell. The SOP model describes well the geometry and 3D shape of the biological particle.
(TIF)

S1 Movie Dynamic force spectroscopy *in silico*: Forced indentation of CCMV capsid along the 2-fold symmetry axis. The movie shows the forced indentation experiment *in silico* on the CCMV shell, in which a compressive force is applied along the 2-fold symmetry axis (side view). The CCMV capsid is positioned on a solid mica surface (small gray colored beads). The cantilever base is moving with the velocity *ν*_*f*_ = 1.0 *μ*m/s perpendicular to the surface of the CCMV shell. As a result, the cantilever tip (large gray colored sphere of radius *R*_*tip*_ = 20 nm) exerts pressure onto the outer surface of the CCMV shell, which undergoes a series of transformations: Heartzian deformation at the early stage of indentation is followed by the bending deformation of the shell side portions, leading to the structural collapse of the capsid. The beams forming the “capsid barrel” fail but not all the same time, which demonstrates the stochastic nature of collapse transitions in vertical beams. Also, formation of small cracks gradually developing into structural damage is clearly observed. The movie stops when the indentation depth *X* reaches *X* = 20 nm. The duration of the indentation experiment is ∼40 ms and the length of the movie is ∼33 s (the movie is played ∼825 times slower than the experiment).
(MPEG)
