# Supplementary material for: Fluctuating Nonlinear Spring Model of Mechanical Deformation of Biological Particles
Source: PLoS Comput Biol. 2016 Jan 28;12(1):e1004729. doi: 10.1371/journal.pcbi.1004729 (PMC4731076; doi:10.1371/journal.pcbi.1004729)
Supplement: S1 Text — (PDF) [file pcbi.1004729.s001.pdf]

Text S1. Self Organized Polymer (SOP) model of a virus particle.

Olga Kononova[1,2], Joost Snijder[3], Yaroslav Kholodov[2,4], Kenneth A. Marx[1], Gijs J. L. Wuite[3], Wouter H. Roos[3,*], Valeri Barsegov[1,2,*].

1 Department of Chemistry, University of Massachusetts, Lowell, MA 01854, USA
2 Moscow Institute of Physics and Technology, Moscow Region, 141700, Russia
3 Natuur- en Sterrenkunde and LaserLab, Vrije Universiteit, 1081 HV Amsterdam, The Netherlands
4 Institute of Computer Aided Design Russian Academy of Science, Moscow, 123056, Russia

The SOP model of the polypeptide chain was originally designed to address the mechanical properties of proteins (see Refs. [24, 25, 36, 37] the main text). The model has been applied to a variety of biological systems [1–3] and Refs. [15] in main text. In this work, the SOP model has been used to describe each protein subunit forming a virus capsid.

In the topology-based SOP model (Fig. S5), each amino acid residue is represented by a single interaction center described by the C_α-atom, and the protein backbone is represented by a collection of the C_α-C_α covalent bonds with the peptide bond length distance of $a = 3.8$ Å. The potential energy function U_{SOP} specified in terms of the coordinates of the C_α-atoms $\{r_i\} = r_1, r_2, \ldots, r_M$ (M is the total number of residues) is given by:

$$U_{SOP} = U_{FENE} + U_{NB}^{ATT} + U_{NB}^{REP} \tag{S1}$$

In Eq.(S1), the first term is the finite extensible nonlinear elastic (FENE) potential:

$$U_{FENE} = -\sum_{i=1}^{M-1} \frac{kR_0}{2} \log\left(1 - \frac{(r_{i,i+1} - r_{i,i+1}^0)^2}{R_0^2}\right) \tag{S2}$$

where k=14 N/m is the spring constant, and the tolerance in the change of the covalent bond distance is R_0=2 Å. The FENE potential describes the backbone chain connectivity. The distance between the next-neighbor residues i and $i+1$, is $r_{i,i+1}$, and $r_{i,i+1}^0$ is its value in the native structure. To account for the non-covalent (non-bonded) interactions that stabilize the native state, we use the Lennard-Jones potential:

$$U_{NB}^{ATT} = \sum_{i,j=i+3}^{M-3} \varepsilon_h \left[\left(\frac{r_{ij}^0}{r_{ij}}\right)^{12} - 2\left(\frac{r_{ij}^0}{r_{ij}}\right)^{6}\right] \Delta_{ij} \tag{S3}$$

In Eq.(S3), we assume that if the non-covalently linked residues i and j ($|i-j| > 2$) are within the cut-off distance of 8 Å in the native state, then $\Delta_{ij} = 1$; $\Delta_{ij} = 0$ otherwise. The value of ε_h quantifies the strength of the non-bonded interactions. The non-native (non-bonded) interactions are treated as repulsive:

$$U_{NB}^{REP} = \sum_{i,j=i+2}^{M-2} \varepsilon_r \left(\frac{\sigma_r}{r_{ij}}\right)^{6} + \sum_{i,j+i+3}^{M-3} \varepsilon_r \left(\frac{\sigma_r}{r_{ij}}\right)^{6} (1 - \Delta_{ij}) \tag{S4}$$

In Eq.(S4), a constraint is imposed on the bond angle between the residues i, $i+1$, and $i+2$ by including the repulsive potential with parameters $\varepsilon_l = 1$ kcal/mol and $\sigma_l = 3.8$ Å. These define the strength and the range of the repulsion. In the SOP model, parameter ε_h sets the energy scale. This parameter is estimated based on the results of all-atom MD simulations of the virus particle at equilibrium.

The dynamics of the virus system is obtained by solving numerically the Langevin equations of motion for each particle position r_i in the over-damped limit:

$$\eta \frac{dr_i}{dt} = -\frac{\partial U_i(r_i)}{\partial r_i} + g_i(t) \quad \text{(S5)}$$

In Eq.(S5), $U_i(r_i)$ is the total potential energy, which accounts for the biomolecular interactions (U_{SOP}) and interactions of particles with the indenting object — spherical tip (U_{tip}; see Eq.(16) in main text). Also, in Eq.(S5) $g_i(t)$ is the Gaussian distributed zero-average random force, and η is the friction coefficient. To generate the Brownian dynamics, the equations of motion for each C_α-atom are propagated with the time step $\Delta t = 0.08\tau_H$, where $\tau_H = \zeta\varepsilon_h\tau_L/k_BT$ ($\Delta t = 20$ ps for CCMV). Here, $\tau_L = (ma^2/\varepsilon_h)^{1/2} = 3$ ps, $\zeta = 50.0$ is the dimensionless friction constant for an amino acid residue in water ($\eta = \zeta m/\tau_L$), $m \approx 3 \times 10^{22}$ g is the residue mass, and T is the absolute temperature [4, 5]. To perform simulations of nanoindentation of a virus particle, we set T to room temperature and use the bulk water viscosity, which corresponds to the friction coefficient $\eta = 7.0 \times 10^5$ pN ps/nm.

References

[1] Zhang Z, Thirumalai D. Dissecting the kinematics of the kinesin step. Structure. 2012;20:628–640.

[2] Hyeon C, Onuchic JN. Mechanical control of the directional stepping dynamics of the kinesin motor. Proc Natl Acad Sci USA. 2007;104:17382–17387.

[3] Zhmurov A, Brown AEX, Litvinov RI, Dima RI, Weisel JW, Barsegov V. Mechanism of fibrin(ogen) forced unfolding. Structure. 2011;19(11):1615–1624.

[4] Barsegov V, Klimov D, Thirumalai D. Mapping the energy landscape of biomolecules using single molecule force correlation spectroscopy: Theory and applications. Biophys J. 2006;90:3827–3841.

[5] Zhmurov A, Dima RI, Barsegov V. Order statistics theory of unfolding of multimeric proteins. Biophys J. 2010;99(6):1959–1968.
