# Supplementary material for: Fluctuating Nonlinear Spring Model of Mechanical Deformation of Biological Particles
Source: PLoS Comput Biol. 2016 Jan 28;12(1):e1004729. doi: 10.1371/journal.pcbi.1004729 (PMC4731076; doi:10.1371/journal.pcbi.1004729)
Supplement: S2 Text — (PDF) [file pcbi.1004729.s002.pdf]

Text S2. Method of Lagrange multipliers.

Olga Kononova[1,2], Joost Snijder[3], Yaroslav Kholodov[2,4], Kenneth A. Marx[1], Gijs J. L. Wuite[3], Wouter H. Roos[3,*], Valeri Barsegov[1,2,*].

1 Department of Chemistry, University of Massachusetts, Lowell, MA 01854, USA
2 Moscow Institute of Physics and Technology, Moscow Region, 141700, Russia
3 Natuur- en Sterrenkunde and LaserLab, Vrije Universiteit, 1081 HV Amsterdam, The Netherlands
4 Institute of Computer Aided Design Russian Academy of Science, Moscow, 123056, Russia

To study the dynamical changes in x_H (Hertzian deformation) and x_b (beam-bending deformation) and their contribution to the total deformation $X{=}x_H + x_b$ (see Fig. 4 in the main text), we employed the method of Lagrange multipliers [1]. This method allows us to find the values of x_H and x_b that minimize the total deformation force, $F(x_H, x_b){=}\ k_H x_H^{3/2}{+}K_b x_b s(x_b)$ (see *the inset* Fig. 4 in main text), subject to the constraint: $X{=}x_H{+}x_b$. To that end, we constructed the Lagrange function

$$\Lambda(x_H, x_b, \lambda) = F(x_H, x_b) + \lambda g(x_H, x_b), \tag{S1}$$

where

$$g(x_H, x_b) = x_H + x_b - X \tag{S2}$$

Here λ is the Lagrange multiplier. By calculating the partial derivatives of $\Lambda(x_H, x_b, \lambda)$ with respect to each of the two variables x_H and x_b, we obtained equations of the form

$$\nabla_{x_H,x_b} F(x_H, x_b) = -\lambda \nabla_{x_H,x_b} g(x_H, x_b) \tag{S3}$$

Next, by eliminating λ we arrived at the system of two equations:

$$3/2 k_H x_H^{1/2} = K_b s(x_b) + K_b x_b s'(x_b) \tag{S4}$$

$$X = x_H + x_b \tag{S5}$$

For small deformations x_b, we expand the Weibull survival probability $s(x_b){=}\exp[-(K_b x_b/F_b^*)^m]$ in powers of the exponent $z{=}K_b x_b/F_b^*$, and retain the terms up to the first order in z. Then, Eq. (S4) becomes:

$$3/2 k_H x_H^{1/2} - K_b(1 - \left(\frac{K_b x_b}{F_b^*}\right)^m)(1 - m\left(\frac{K_b x_b}{F_b^*}\right)^m) = 0 \tag{S6}$$

Eq. (S5) allows us to eliminate x_H by substituting $x_H{=}X{-}x_b$ into Eq. (S6) above:

$$3/2 k_H (X - x_b)^{1/2} - K_b(1 - \left(\frac{K_b x_b}{F_b^*}\right)^m)(1 - m\left(\frac{K_b x_b}{F_b^*}\right)^m) = 0 \tag{S7}$$

Simplifying Eq. (S7) and grouping terms of the same power in x_b, we arrive at the following polynomial equation:

$$a_1 x_b^{4m} + a_2 x_b^{3m} + a_3 x_b^{2m} + a_4 x_b^m + a_5 x_b + a_6 = 0 \tag{S8}$$

with the following constant coefficients:

$$
\begin{aligned}
a_1 &= m^2 K_b^2 \left(\frac{K_b}{F_b^*}\right)^{4m} \\
a_2 &= -2m(1+m)K_b^2 \left(\frac{K_b}{F_b^*}\right)^{3m} \\
a_3 &= (1+4m+m^2)K_b^2 \left(\frac{K_b}{F_b^*}\right)^{2m} \\
a_4 &= -2(1+m)K_b^2 \left(\frac{K_b}{F_b^*}\right)^{m} \\
a_5 &= \frac{9}{4}k_H^2 \\
a_6 &= K_b^2 - \frac{9}{4}k_H^2 X
\end{aligned}
\qquad \text{(S9)}
$$

Eq. (S8) can be solved numerically (for example, using Mathematica software) for a given set of parameters k_H, K_b, F_b^*, and m, and for each specified value of the total deformation X. The obtained numerical solution for x_H and x_b can then be substituted into the expression for $F(x_H, x_b)$ (Eq. (14) in the main text).

References

[1] McQuarrie DA. Statistical Mechanics. University Science Books; 2000.
