# Supplementary material for: Fluctuating Nonlinear Spring Model of Mechanical Deformation of Biological Particles
Source: PLoS Comput Biol. 2016 Jan 28;12(1):e1004729. doi: 10.1371/journal.pcbi.1004729 (PMC4731076; doi:10.1371/journal.pcbi.1004729)
Supplement: S3 Text — (PDF) [file pcbi.1004729.s003.pdf]

Text S3. Estimation of the thickness of TrV and AdV with encapsulated genome.

Olga Kononova[1,2], Joost Snijder[3], Yaroslav Kholodov[2,4], Kenneth A. Marx[1], Gijs J. L. Wuite[3], Wouter H. Roos[3,*], Valeri Barsegov[1,2,*].

1 Department of Chemistry, University of Massachusetts, Lowell, MA 01854, USA
2 Moscow Institute of Physics and Technology, Moscow Region, 141700, Russia
3 Natuur- en Sterrenkunde and LaserLab, Vrije Universiteit, 1081 HV Amsterdam, The Netherlands
4 Institute of Computer Aided Design Russian Academy of Science, Moscow, 123056, Russia

When a virus particle is empty (i.e. it does not contain genomic material) the thickness of the beams is equal to the virus shell thickness. The capsid thickness with encapsulated genome due to DNA/RNA packing can be estimated assuming that DNA/RNA molecules are evenly distributed on the inner capsid surface. The volume occupied by the genome considered to form a long cylindrical tube is given by $V_{gen}=\pi R_{gen}^2 L_{gen}$, where L_{gen} is the genome length and R_{gen}=1.0 nm and 0.5 nm is the radius of cross-sectional area of the dsDNA molecule (for AdV) and ssRNA molecule (for TrV), respectively. The inner volume of the virus particle, $V_{in}=V_{gen}+V_{emp}$, is the sum of the volume occupied by the genome (V_{gen}) and the volume of the empty space (V_{emp}). Here, $V_{in}=4/3\pi R_{in}^3$ and $V_{emp}=4/3\pi(R_{in}-r_{gen})^3$, where $R_{in}=R_{par}-r$ is the inner radius of the particle shell, r is the shell thickness, and r_{gen} is the increase in shell thickness due to encapsulated genome. This allows us to express r_{gen} as $r_{gen}=R_{in}-(R_{in}^3-3/4R_{gen}^2L_{gen})^{1/3}$. For the AdV shell, R_{in}=36 nm and L_{gen}=12 μm [1]; hence, r_{gen}=2.5 nm. For the TrV shell, R_{in}=11 nm and L_{gen}=3.06 μm (Ref. [12] in main text); hence, r_{gen}=1.9 nm. These values of r_{gen} for AdV and TrV shells were used to estimate E_b (Table 1 in main text).

References

[1] San Martin C. Latest insights on Adenovirus structure and assembly. Viruses. 2012;4:847.
