# Supplementary material for: Fluctuating Nonlinear Spring Model of Mechanical Deformation of Biological Particles
Source: PLoS Comput Biol. 2016 Jan 28;12(1):e1004729. doi: 10.1371/journal.pcbi.1004729 (PMC4731076; doi:10.1371/journal.pcbi.1004729)
Supplement: S4 Text — (PDF) [file pcbi.1004729.s004.pdf]

Text S4. SOP model parameterization for CCMV shell.

Olga Kononova[1,2], Joost Snijder[3], Yaroslav Kholodov[2,4], Kenneth A. Marx[1], Gijs J. L. Wuite[3], Wouter H. Roos[3,*], Valeri Barsegov[1,2,*].

1 Department of Chemistry, University of Massachusetts, Lowell, MA 01854, USA
2 Moscow Institute of Physics and Technology, Moscow Region, 141700, Russia
3 Natuur- en Sterrenkunde and LaserLab, Vrije Universiteit, 1081 HV Amsterdam, The Netherlands
4 Institute of Computer Aided Design Russian Academy of Science, Moscow, 123056, Russia

In the SOP model (Refs. [36,37] in main text), the parameter ε_h defines the average strength of the non-covalent residue-residue contacts that stabilize the native state of the system (biological particle). The values of ε_h has to be calculated directly using the equilibrium MD simulations of an atomic model of the biological particle in question. We performed the all-atom MD simulations of the atomic structural model of the CCMV shell in implicit solvent (Fig. S1). To obtain accurate parameterization of the SOP model for CCMV, we used the crystal structure of the capsid (Viper Data Base; PDB code: 1CWP [1] with T=3 symmetry) in conjunction with the Solvent Accessible Surface Area (SASA) model of implicit solvation (CHARMM19 force field) [2]. First, we calculated the number of native contacts using a standard cut-off distance between the C_α-atoms of 8.0 Å. The native contacts were divided into the inter-chain contacts and the intra-chain contacts. For the CCMV shell, there are M_{intra}≈20,554 intra-chain contacts that stabilize the native state of the capsid protein, and M_{inter}≈3,405 inter-chain contacts at the interfaces formed by capsid proteins. Next, we calculated the total energy of non-covalent interactions for each contact group. The total energy for the intra-chain contacts is E_{intra}=25,898 kcal/mol and the total energy for the inter-chain contacts is E_{inter}=3,746 kcal/mol. Finally, to obtain the values of parameter ε_h we divided the two numbers for each contact group. For the CCMV shell, we obtained $\varepsilon_{intra}=E_{intra}/M_{intra}=1.26$ kcal/mol and $\varepsilon_{inter}=E_{inter}/M_{inter}=1.1$ kcal/mol. The atomic-level details of biomolecular interactions, contained in these parameters, are then exported into the SOP model based reconstruction of the full CCMV particle (Fig. S5).

References

[1] Speir JA, Munshi S, Wang G, Baker TS, Johnson JE. Structures of the native and swollen forms of cowpea chlorotic mottle virus determined by X-ray crystallography and cryo-electron microscopy. Structure. 1995;3:63–78.

[2] Ferrara P, Apostolakis J, Caflisch A. Evaluation of a fast implicit solvent model for molecular dynamics simulations. Proteins. 2002;46(1):24–33.
