# Supplementary figures and images for: Fluctuating Nonlinear Spring Model of Mechanical Deformation of Biological Particles
Source: PLoS Comput Biol. 2016 Jan 28;12(1):e1004729. doi: 10.1371/journal.pcbi.1004729 (PMC4731076; doi:10.1371/journal.pcbi.1004729)

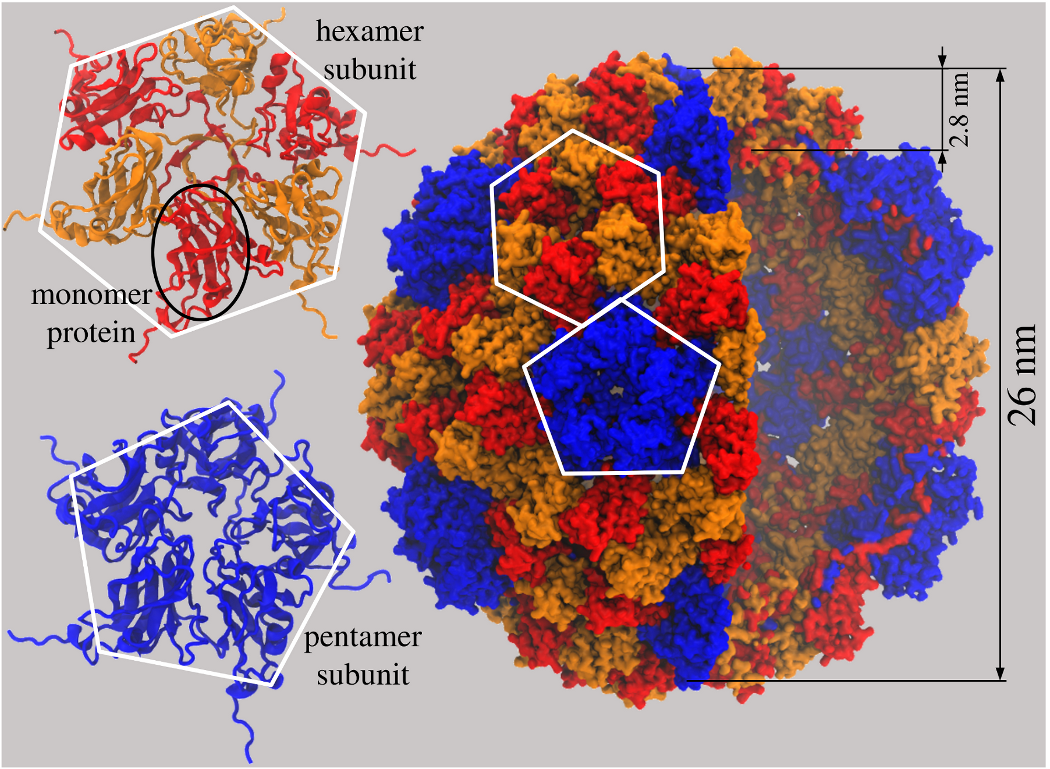

Supplement: S1 Fig — The side view of the CCMV shell is shown on the right. The protein domains forming pentamers are in blue, while the same protein domains in hexamers are in red and orange. The hexamers and pentamers, composed of six and five copies of the same protein chain (circled in the black ellipse), are displayed on the left. The CCMV capsid is a ∼2.8 nm thick shell with a ∼26 nm diameter. (TIFF) [file pcbi.1004729.s005.tiff]

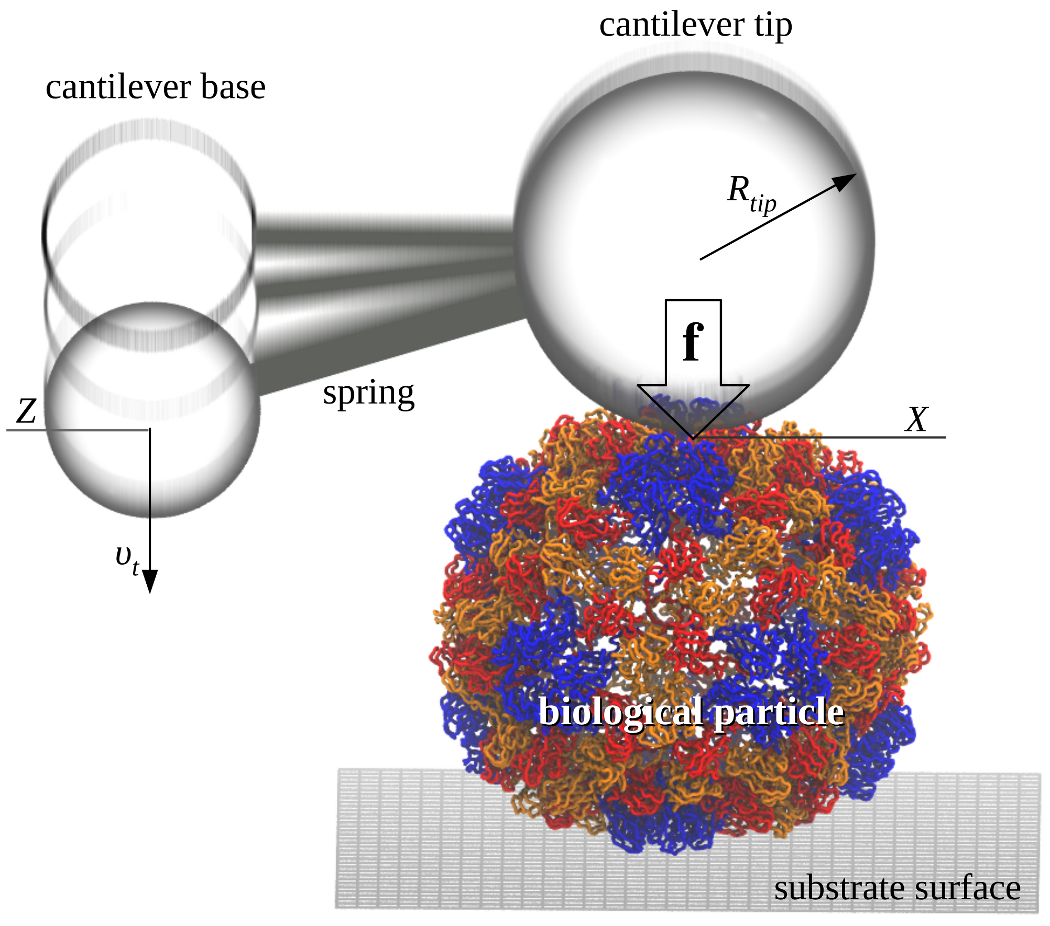

Supplement: S2 Fig — The biological particle (virus shell) is placed on the substrate. The cantilever base (virtual sphere) is moving in the direction perpendicular to the surface of the particle with the constant velocity ν f (force-ramp), which creates a compressive force. The force is transmitted to the cantilever tip (sphere of radius R tip) through the harmonic spring with the spring constant κ. The force exerted on a particle f(t) = r f t (large vertical arrow) ramps up linearly in magnitude with time with the force-loading rate r f = κν f, which mechanically loads the particle. The mechanical response of the particle can be probed by profiling the deformation force (indentation force) F as a function of the cantilever base (piezo-) displacement Z (FZ curve) or as a function of the indentation depth X (FX curve). (TIFF) [file pcbi.1004729.s006.tiff]

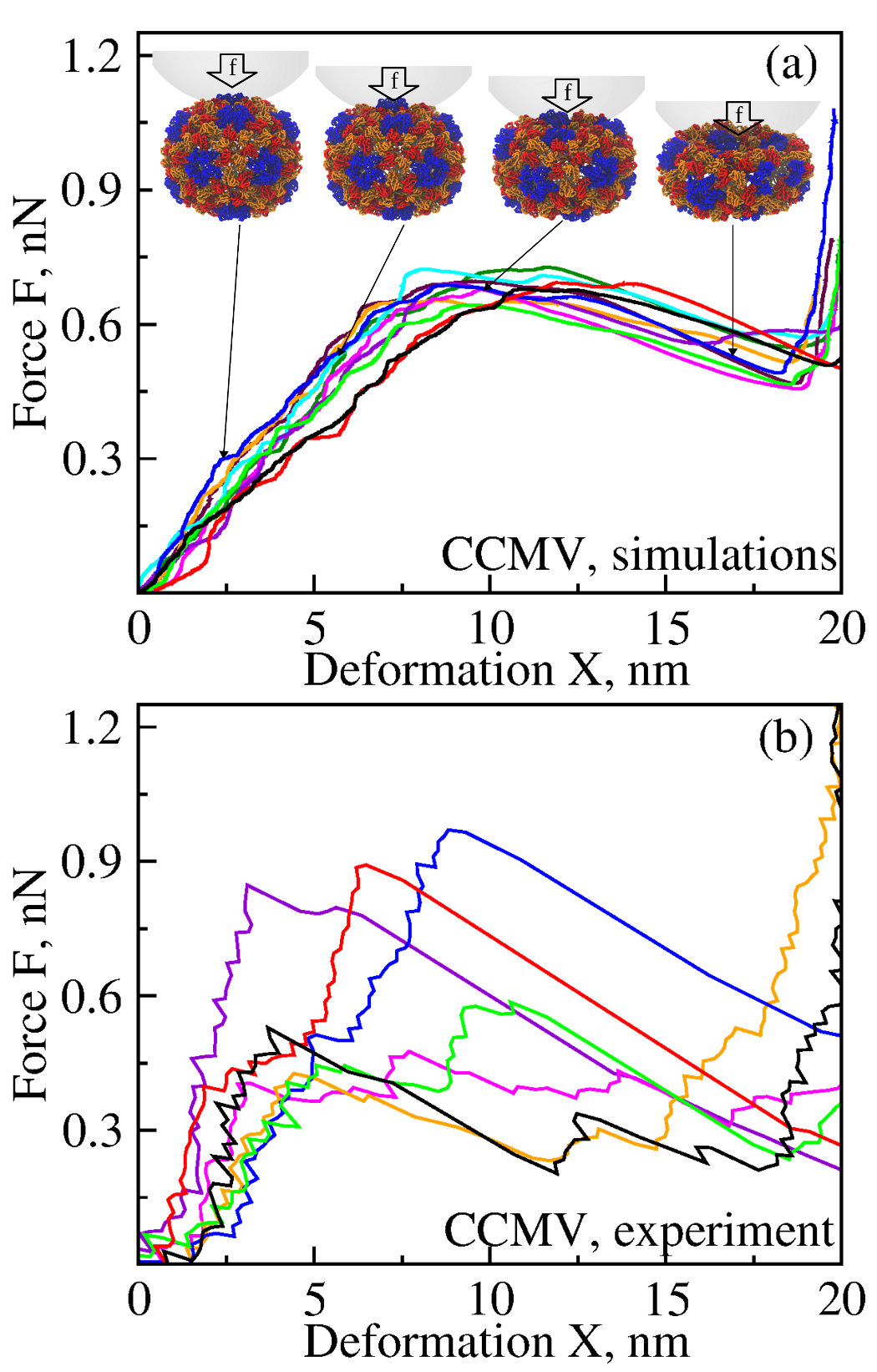

Supplement: S3 Fig — Shown in different colors for clarity are the FX curves obtained using the cantilever tip velocity ν f = 0.06 μm/s (experiment) and ν f = 1.0 μm/s (simulations). In the AFM-based experiments and in simulations of nanoindentation of CCMV, we used the cantilever tip with radius R tip = 20 nm and the spring constant κ = 0.05 N/m. In panel (a), structural snapshots from the left to the right, which correspond to the FX curve shown in blue, display the progress of forced deformation from the native un-deformed state (leftmost structure), to the partially deformed state (middle structures), and finally to the globally collapsed state (rightmost structure). In nanoindentation measurements in silico and in vitro, the cantilever tip indents the capsid in the direction perpendicular to the capsid outer surface (shown by a large vertical arrow). Simulation and experimental data are from Ref. [15] in the main text, please see this publication for exact experimental procedures and results. (TIFF) [file pcbi.1004729.s007.tiff]

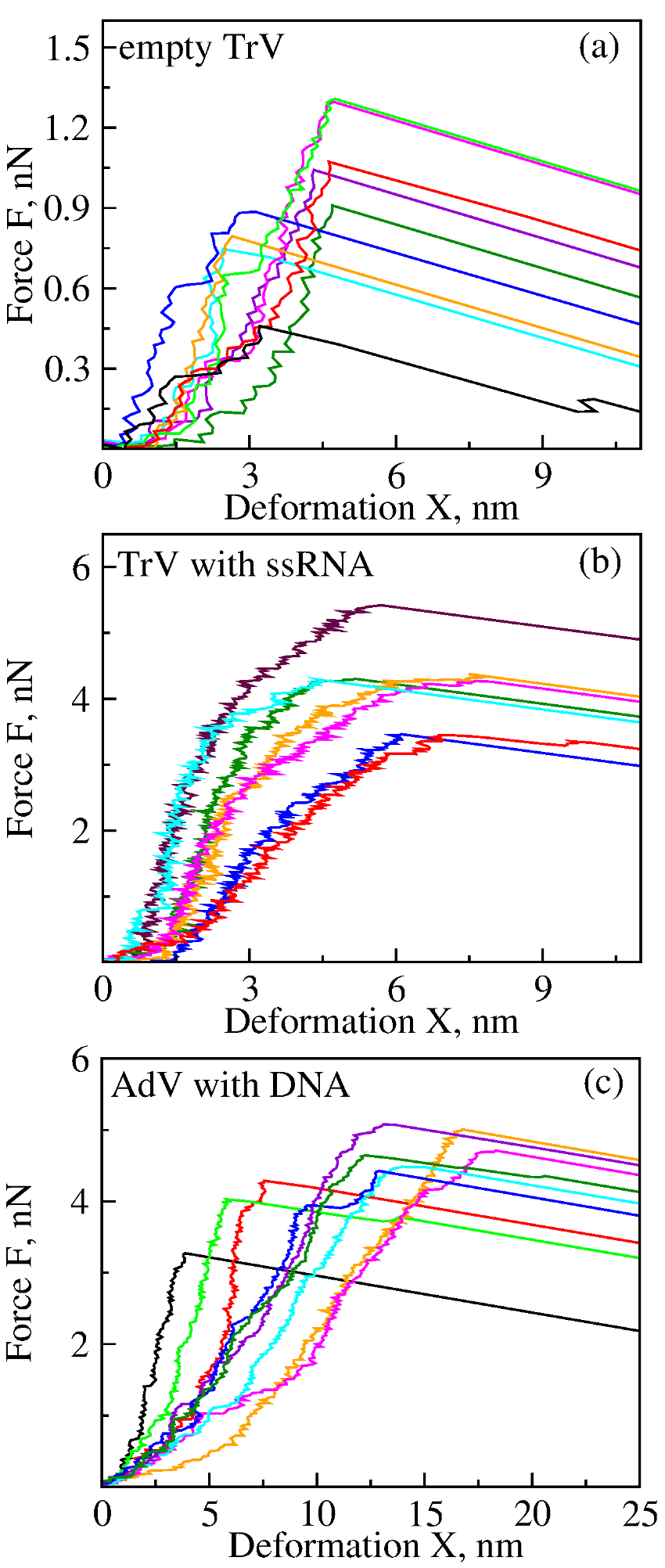

Supplement: S4 Fig — Shown in different colors for clarity are the representative force-deformation spectra. The FX curves for the empty TrV particle were obtained using the cantilever tip velocity ν f = 0.06 μm/s, tip radius R tip = 15 nm, and spring constant κ = 0.056 N/m. The FX curves for the full TrV particle were obtained using ν f = 0.06 μm/s, R tip = 15 nm, and κ = 0.1 N/m. The FX curves for the full AdV particle were obtained using ν f = 0.055 μm/s, R tip = 15 nm, and κ = 0.0524 N/m. Experimental data are from Refs. [8, 12] in the main text, please see these publications for exact experimental procedures and results. (TIF) [file pcbi.1004729.s008.tif]

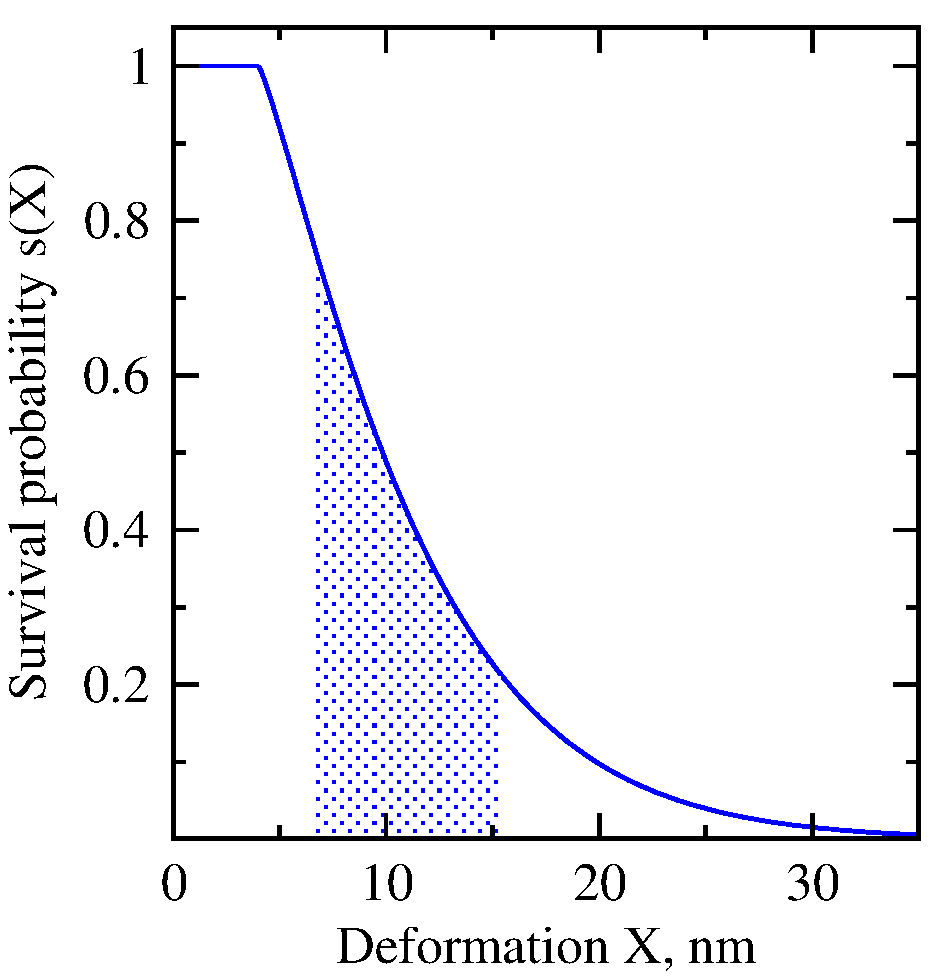

Supplement: S5 Fig — Shown is the curve of s(X) obtained using Eq (16) with FNS model parameters for the empty CCMV particle tested experimentally (see Table 1 in the main text). The shaded area represents the width of the transition range ΔX ≈ 8.0 nm, which compares well with the experimental value of the same quantity ΔX col = 6 nm from statistical analysis of critical deformations (S3b Fig). (TIF) [file pcbi.1004729.s009.tif]

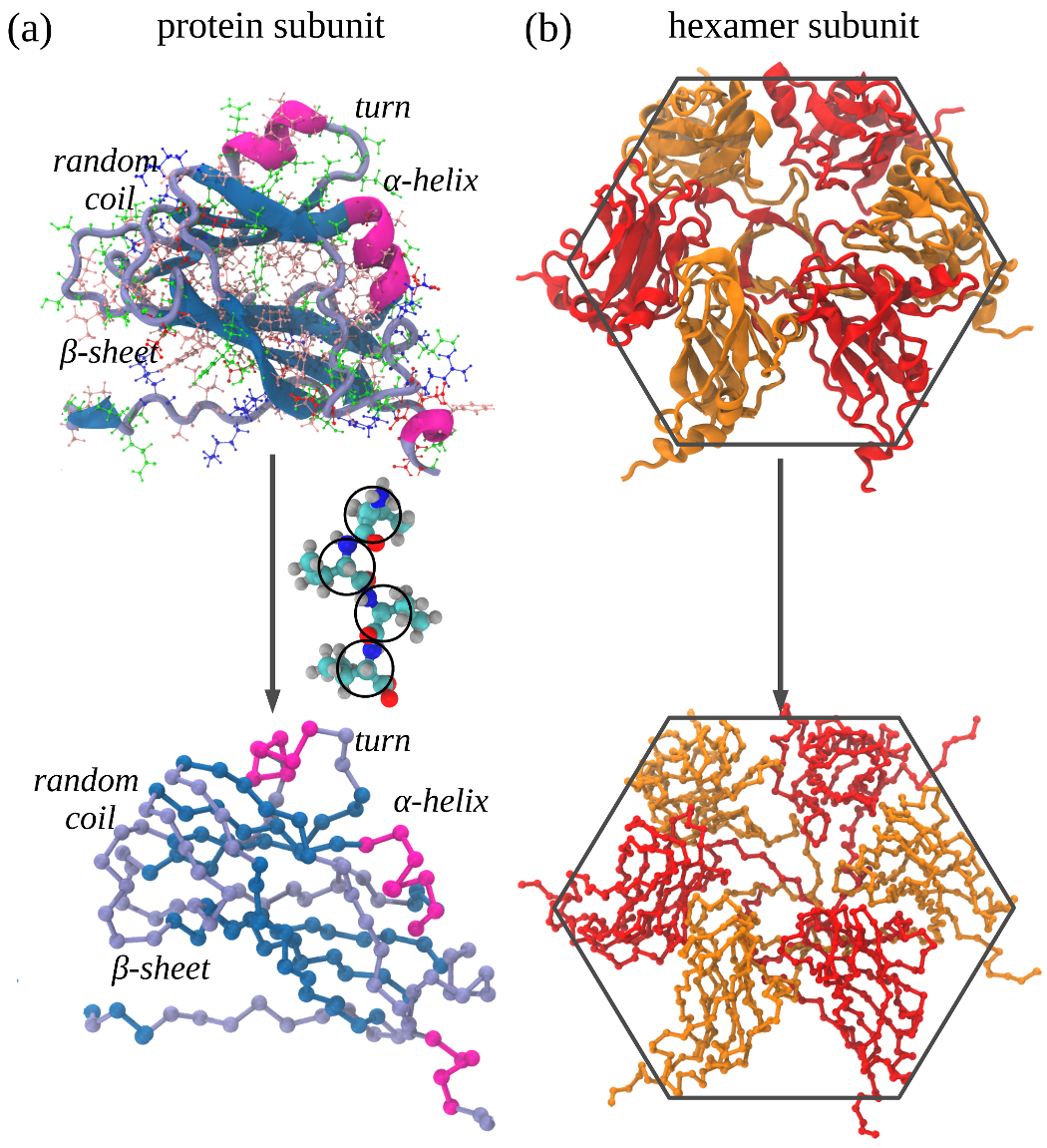

Supplement: S6 Fig — Panel (a) shows coarse-graining of the atomic structure of the protein subunit forming pentamers and capsomers of the CCMV shell (S1 Fig). Each amino acid residue is represented by a spherical bead of an appropriate radius with the coordinates of the C α-atom (black circles). The protein backbone is replaced by a collection of the C α-C α covalent bonds with 3.8 Å bond distance. The potential energy function (see Eq. S1 in S1 Text) describes the interactions between amino acids stabilizing the native state of the protein chain, and the chain connectivity, elongation due to stretching, and self-avoidance. The coarse-graining procedure preserves the secondary structure: α-helices (pink), β-strands and sheets (blue), and random coil and turns (gray). Panel (b) shows the results of coarse-graining of a hexamer. Six identical copies of the same protein monomer (coarse-grained in (a)) form a C α-based model of the hexamer subunit. The hexamers and pentamers are combined to form a coarse-grained reconstruction of the full CCMV shell. The SOP model describes well the geometry and 3D shape of the biological particle. (TIF) [file pcbi.1004729.s010.tif]
